# *Streptococcus salivarius*-derived ilexgenin A alleviates pneumonia through the gut-lung axis

**DOI:** 10.1128/msystems.00731-25

**Published:** 2025-07-30

**Authors:** Xintao Huang, Rong Wu, Xiaoqi Liang, Zhichao Yu, Ping Qin, Ze Wang, Peiheng Guo, Yunong Zeng, Zhengzheng Yan, Wei Xiao, Yun Ma

**Affiliations:** 1 Department of Pharmacy, Clinical Pharmacy, Clinical Pharmacy Center, Nanfang Hospital, Southern Medical University 70570, Guangzhou, China; 2 School of Traditional Chinese Medicine, Southern Medical University 70570, Guangzhou, China; 3 The Tenth Affiliated Hospital (Dongguan People's Hospital), Shenzhen Clinical Medical School, Southern Medical University 826277, Dongguan, Guangzhou, China; 4 Center for Drug Research and Development, Guangdong Provincial Key Laboratory of Advanced Drug Delivery System, Guangdong Pharmaceutical University 71237, Guangzhou, China; 5 Department of Critical Care Medicine, The Third Affiliated Hospital of Southern Medical University 572489, Guangzhou, China; University of California San Diego 8784, La Jolla, California, USA

**Keywords:** *Pseudomonas aeruginosa*, pneumonia, gut microbiota, *Streptococcus salivarius*, ilexgenin A, TLR4

## Abstract

The alteration of gut microbiota during critical illness is associated with adverse clinical outcomes. This connection between intestinal dysbiosis and poor outcomes has prompted the idea that restoring healthy microbial communities could offer a novel approach to life-support treatment for patients with severe pneumonia. In this study, using 16S rRNA sequencing and fecal microbiota transplantation (FMT), we demonstrated that alterations in intestinal microbiota structure during pneumonia exacerbate disease outcomes. A notable feature of these alterations is the reduction in the relative levels of *Streptococcus salivarius* (*S. salivarius*). In combination with metabolomics analysis, we found that the administration of *S. salivarius* increased the level of ilexgenin A (IA) in mice, which enhances the resistance of mice to *Pseudomonas aeruginosa* (*P. aeruginosa*)-induced pneumonia. Mechanistically, IA regulates lipopolysaccharide-induced overexpression of macrophage inflammation through Toll-like receptor 4 (TLR4)-mediated NF-κB and MAPK signaling pathways. Our findings reveal the role of the microbial–immune axis in pneumonia, highlighting the potential of *S. salivarius* and IA in providing promising treatment strategies for pneumonia.

**IMPORTANCE:**

One of the major challenges faced by the clinical microbiome research community is to convert the connections between dysbiosis and negative clinical outcomes into rationalized and targeted therapeutic interventions. In the present work, 30 fecal samples from pneumonia and non-pneumonia patients were subjected to FMT and 16S rRNA analysis. The results revealed that a characteristic feature of gut microbiota dysbiosis in pneumonia hosts is the reduction of *S. salivarius*. Supplementation with *S. salivarius* can effectively enhance the resistance of mice to *P. aeruginosa* pneumonia. Moreover, we confirmed the anti-inflammatory effects of IA derived from *S. salivarius* both *in vivo* and *in vitro*. Thus, these findings enhance our understanding of how gut microbiota influences the outcomes of pneumonia and underscore the potential of *S. salivarius* as a precision microbial therapeutic for combating pneumonia.

## INTRODUCTION

Pneumonia is an inflammatory condition of the lungs caused by various etiological agents, with bacterial pneumonia being the most common form (1, 2). The crude mortality rate for nosocomial pneumonia can be as high as 70% (3). Several reports have estimated that one-third to half of ventilator-associated pneumonia (VAP)-related deaths are directly due to infection (4, 5). In the context of hospital-acquired pneumonia and VAP, *Pseudomonas aeruginosa* is frequently encountered, often leading to pneumonia and sepsis in intensive care unit (ICU) patients (6, 7). Infections caused by these organisms are notoriously difficult to treat and often result in therapeutic failure due to their severe inflammatory responses and multidrug resistance mechanisms, such as efflux pumps, β-lactamases, and mutational resistance (8). Therefore, novel therapeutic approaches to address the challenges of pneumonia are urgently needed.

The relationship between gut dysbiosis and adverse outcomes in critically ill patients, along with the feasibility of modifying the gut microbiota, has generated significant interest in microbiota interventions as an adjunctive treatment for critical illnesses (9). Current research is focused on unraveling the cellular and molecular links between gut dysbiosis and infections and identifying opportunities for therapeutic modulation of host-microbe interactions (10–12). Our previous research showed that formononetin, secreted by the intestinal bacterium *Parabacterioides merdae*, can regulate macrophage pyroptosis, thereby affecting the susceptibility of pregnant women to sepsis (13). Mechanistically, restoring key commensals may reinstate microbiota-immune balance, enhancing host defense against infections. Evidence from epidemiological studies, clinical trials, and animal models also strongly supports the role of the gut-lung axis in pneumonia (14–16). However, our understanding of the underlying principles is still insufficient, particularly regarding the regulatory mechanisms of beneficial symbionts in the context of pneumonia.

In this study, we investigated the role of *Streptococcus salivarius* in combating pneumonia induced by *P. aeruginosa*. Our findings demonstrate that *S. salivarius* metabolically derives IA, which serves as a key mediator of its anti-pneumonia effects and exhibits significant anti-inflammatory properties both *in vivo* and *in vitro*. Furthermore, using *TLR4^-/-^* bone marrow-derived macrophages (BMDMs), we elucidated the role of the TLR4 signaling pathway in mediating the anti-inflammatory effects of IA. Thus, our results suggest that *S. salivarius*-derived IA alleviates pneumonia by modulating macrophage inflammation through the TLR4 signaling pathway, offering a promising therapeutic strategy for pneumonia caused by *P. aeruginosa*.

## RESULTS

### Alteration of the gut microbiota modulates susceptibility to pneumonia

**Fig 1:**
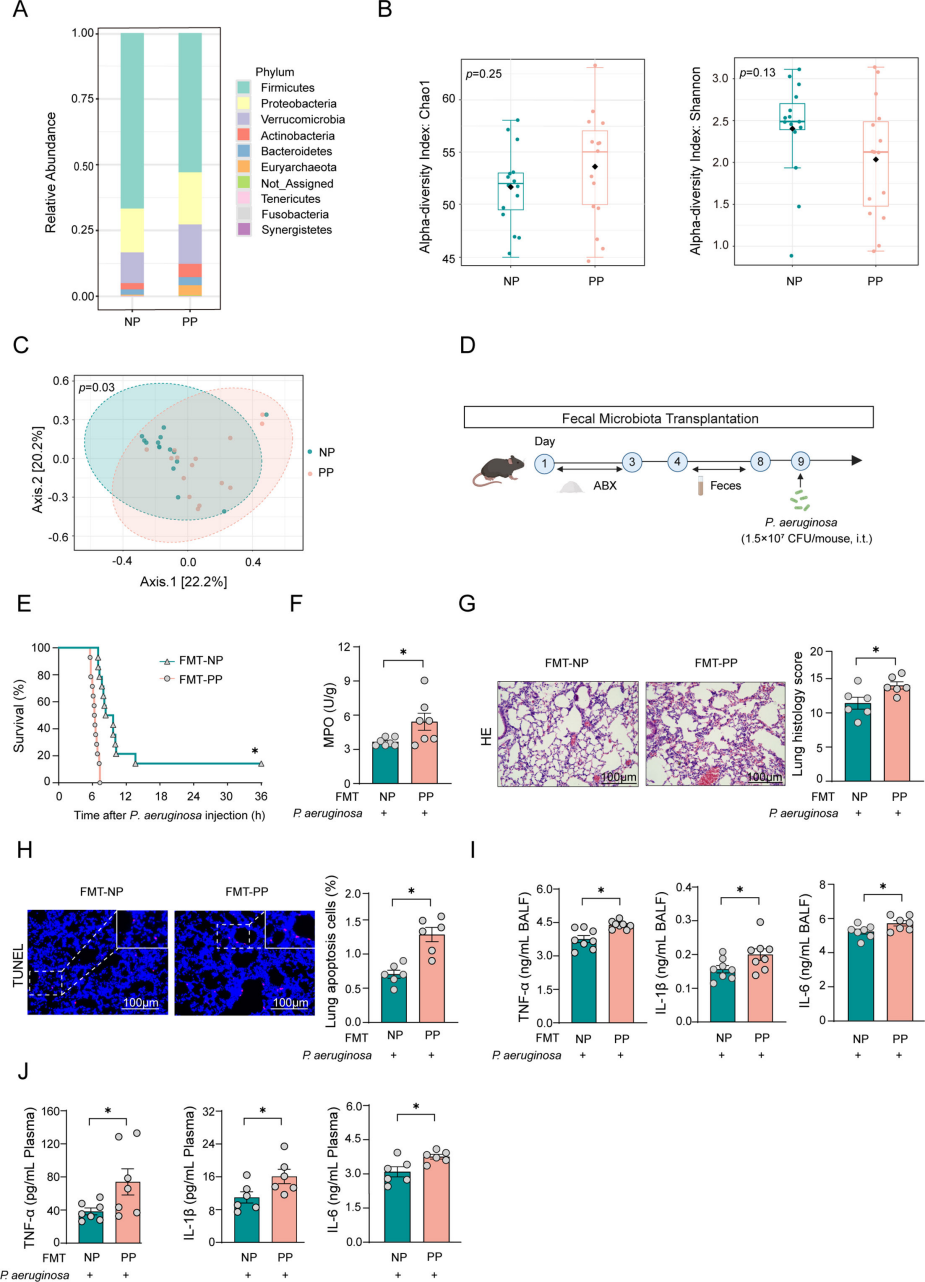
Alteration of the gut microbiota modulates susceptibility to pneumonia**.** (A) Relative abundance of bacteria at the phylum level in non-pneumonia patients and pneumonia patients. *n* = 15. (B) Chao 1 and Shannon’s diversity index in non-pneumonia patients and pneumonia patients. *n* = 15. (C) PCoA of the gut microbiota in non-pneumonia patients and pneumonia patients based on the Bray–Curtis distance. *n* = 15. (D) FMT experiment flowchart. Mice were treated with a mixture of ABX (100 mg/kg vancomycin, 200 mg/kg neomycin sulfate, 200 mg/kg metronidazole, and 200 mg/kg ampicillin), followed by fecal microbiota solution (25 mg/mouse). (E) Survival curve of FMT mice subjected to *P. aeruginosa* infection (5 × 10^7^ CFU per mouse, i.t.). *n* = 15. (F) MPO activity in lung tissue from FMT mice. Tissues were harvested when the first mouse became moribund around 6 h after infection. *n* = 7. (G, H) H&E and TUNEL staining were performed to quantify lung injury and cell death percentages in the lung. *n* = 6. (I) Quantification of cytokines in BALF from FMT mice after *P. aeruginosa* infection. *n* = 6. (J) Quantification of cytokines in plasma from FMT mice after *P. aeruginosa* infection. *n* = 6. Scale bar, 100 µm. Data are shown as mean ± SEM. Comparisons were assessed by two-tailed unpaired *t*-test. The survival rates of septic mice were analyzed using the Kaplan-Meier method with log-rank tests. *P < 0.05.

We initially collected fecal specimens from non-pneumonia patients (NP) and pneumonia patients (PP) in the hospital for 16S rRNA sequencing analysis to investigate differences in the gut microbiota. The results indicated that in comparison to the NP group, the gut microbiota composition at the phylum level was significantly altered in pneumonia patients, characterized by a reduction in the relative abundance of Firmicutes and an increase in the relative abundance of Proteobacteria (Fig. 1A). Through α-diversity analysis, we observed a trend indicating increased bacterial abundance and reduced diversity in the gut microbiota of pneumonia patients. However, these differences were not statistically significant (Fig. 1B).

Principal coordinates analysis (PCoA) at the feature level revealed significant discrepancies in the β-diversity of the gut microbiota, indicating distinct microbial profiles between the NP group and the PP group (Fig. 1C). To assess whether these variations in microbial composition influence susceptibility to pneumonia, fecal microbiota transplantation (FMT) experiments were performed (Fig. 1D). The results demonstrated that fecal transplantation from pneumonia patients accelerated the mortality rate of mice infected with *P. aeruginosa* (Fig. 1E). Biochemical assays revealed that mice receiving fecal microbiota transplantation from pneumonia patients exhibited higher myeloperoxidase (MPO) levels after infection with *P. aeruginosa* (Fig. 1F). Pathological assessments further indicated that FMT-PP mice exhibited significantly more severe organ damage and cellular apoptosis compared with FMT-NP mice (Fig. 1G and H). Additionally, the levels of the inflammatory cytokines IL-1β, IL-6, and TNF-α in bronchoalveolar lavage fluid (BALF) and plasma were significantly elevated in mice receiving FMT from patients with pneumonia (Fig. 1I and J). In summary, these findings suggest that the microbial composition in pneumonia patients undergoes profound alterations and that susceptibility to infectious pneumonia can be conferred through the gut microbiota.

### Alterations of *S. salivarius* in the gut mediate susceptibility to pneumonia

**Fig 2:**
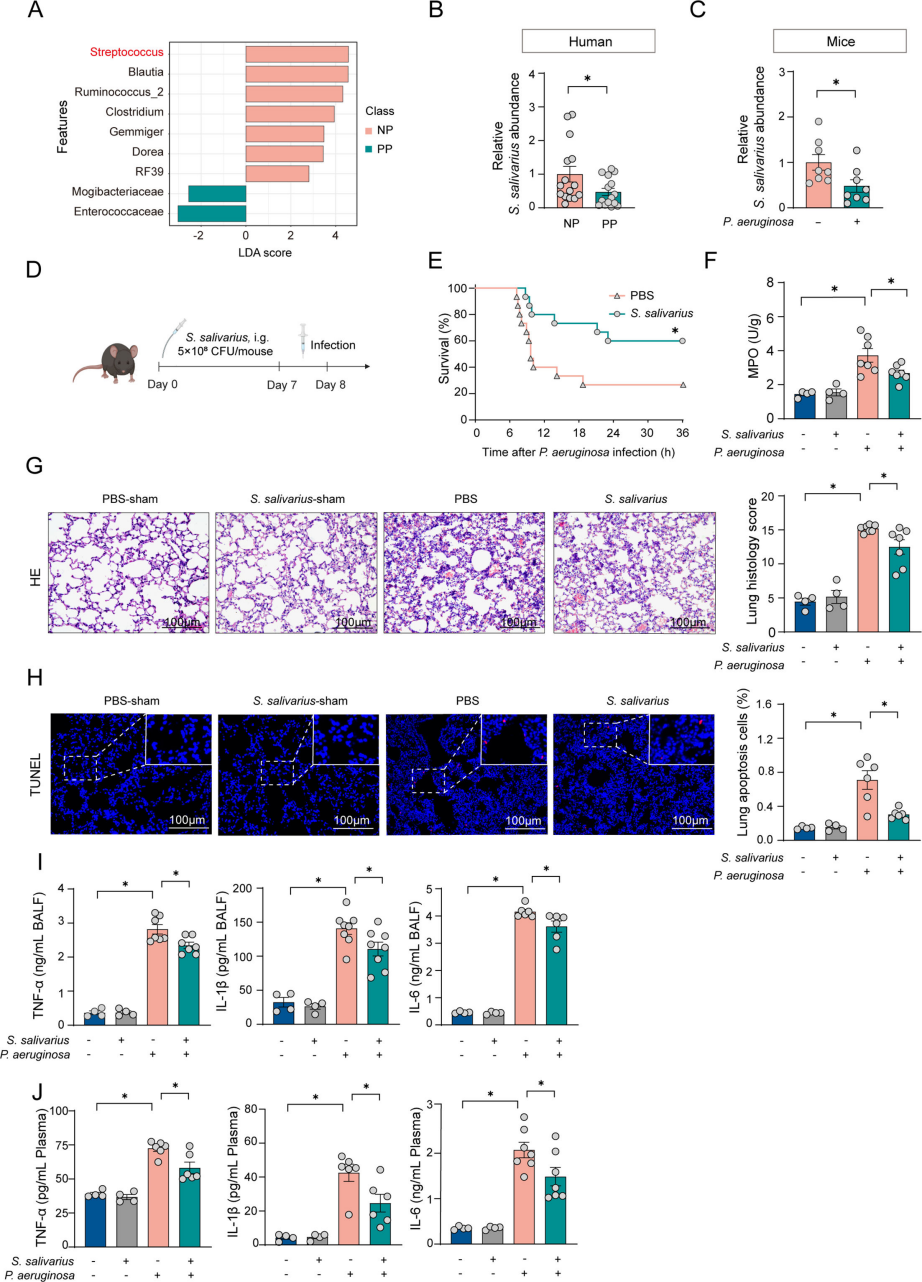
Alterations of *S. salivarius* in the gut mediate susceptibility to pneumonia. (A) Linear discriminant analysis effect size (LEfSe) of the gut microbiota in NP group and PP group. *n* = 15. (B) Relative abundance of *S. salivarius* in NP group and PP group. *n* = 15. (C) Relative abundance of *S. salivarius* in mice with or without *P. aeruginosa* infection. *n* = 8. (D) Mice were gavaged with 5 × 10^8^ CFU of *S. salivarius* for 7 days. Mice were infected with *P. aeruginosa* on the 8th day. (E) The survival curve of infected mice subjected to pretreatment with *S. salivarius*. *n* = 15. (F) MPO activity in the lungs of infected mice with or without *S. salivarius* pretreatment. (G, H) H&E and TUNEL staining were performed to quantify lung injury and cell death percentages in *S. salivarius*-pretreated mice. *n* = 6. (I) Quantification of cytokines in the BALF from infected mice subjected to pretreatment with *S. salivarius*. *n* = 6. (J) Quantification of cytokines in the plasma from infected mice subjected to pretreatment with *S. salivarius*. *n* = 6. Scale bar, 100 µm. Data were shown as mean ± SEM. Comparisons were assessed using a one-way ANOVA. The survival rates of septic mice were analyzed using the Kaplan-Meier method with log-rank tests, *P < 0.05.

To further elucidate the mechanism by which the gut microbiota affects susceptibility to pneumonia, we performed linear discriminant analysis effect size (LEfSe) to compare the PP group and NP group. This analysis identified the *Streptococcus* genus as exhibiting the most significant differences between the two groups, with a pronounced decreasing trend observed in the PP group (Fig. 2A). Subsequently, we noted a reduction in the relative abundance of *S. salivarius* in the pneumonia group, which was corroborated by qRT-PCR assessments of fecal specimens from both human and mouse sources (Fig. 2B and C). Thus, we concentrated our investigation on the association between *S. salivarius* and pneumonia.

To exclude the effect of *S. salivarius* on energy metabolism, we first measured food intake and body weight over the experiment period and found no differences between the control and *S. salivarius*-treated mice (Fig. S1A and B). Subsequently, we administered *S. salivarius* orally to mice to assess the treatment effect (Fig. 2D). The results demonstrated that oral administration of *S. salivarius* significantly improved the survival rate of mice infected with *P. aeruginosa*. Similar protective effects were observed in mice infected with *Staphylococcus aureus* (*S. aureus*) and *Klebsiella pneumoniae* (*K. pneumoniae*)-induced pneumonia (Fig. 2E and Fig. S1C and D). Biochemical indicators showed that compared with the control group, the lung MPO levels in mice infected with *P. aeruginosa* were significantly increased, whereas treatment with *S. salivarius* reduced abnormal MPO levels (Fig. 2F). Histological analysis showed that the control group mice displayed no signs of lung damage, whereas the *P. aeruginosa* infection group exhibited extensive inflammatory cell infiltration, thickening of alveolar walls, and severe hemorrhage. In contrast, lung damage was reversed in the *S. salivarius* group (Fig. 2G). Additionally, TUNEL staining demonstrated a significant increase in lung cell death due to *P. aeruginosa* infection compared with the control group, whereas *S. salivarius* reduced this abnormal increase (Fig. 2H). In our study, we observed a significant elevation in the levels of inflammatory cytokines IL-1β, IL-6, and TNF in BALF and plasma following *P. aeruginosa* infection in mice. Notably, intervention with *S. salivarius* effectively attenuated these elevated cytokine levels (Fig. 2I and J). Additionally, we evaluated the colonization efficacy of *S. salivarius* within the gastrointestinal tract. qRT-PCR analyses indicated that the gavage of *S. salivarius* results in a significant elevation of its relative abundance in the cecum (Fig. S1E). Meanwhile, as expected, recipient mice that received fecal transplants from donors treated with *S. salivarius* showed increased survival rates and reduced tracheal damage and inflammation levels after *P. aeruginosa* infection, demonstrating resistance to the infection (Fig. S1F through K). Collectively, these results suggest that intervention with *S. salivarius* in the gut confers a protective effect against pneumonia induced by *P. aeruginosa* infection in mice.

### *S. salivarius* induces the biosynthesis of IA

To further elucidate the mechanisms by which *S. salivarius* influences pneumonia, we conducted an analysis of the 16S rRNA gene sequences obtained from fecal samples of mice gavaged with *S. salivarius*. We found that *S. salivarius* does not induce significant alterations in the richness or composition of the microbial community (Fig. S2A through C). Subsequently, we hypothesized that the metabolic byproducts of *S. salivarius* might serve as potential mediators of its effects.

**Fig 3:**
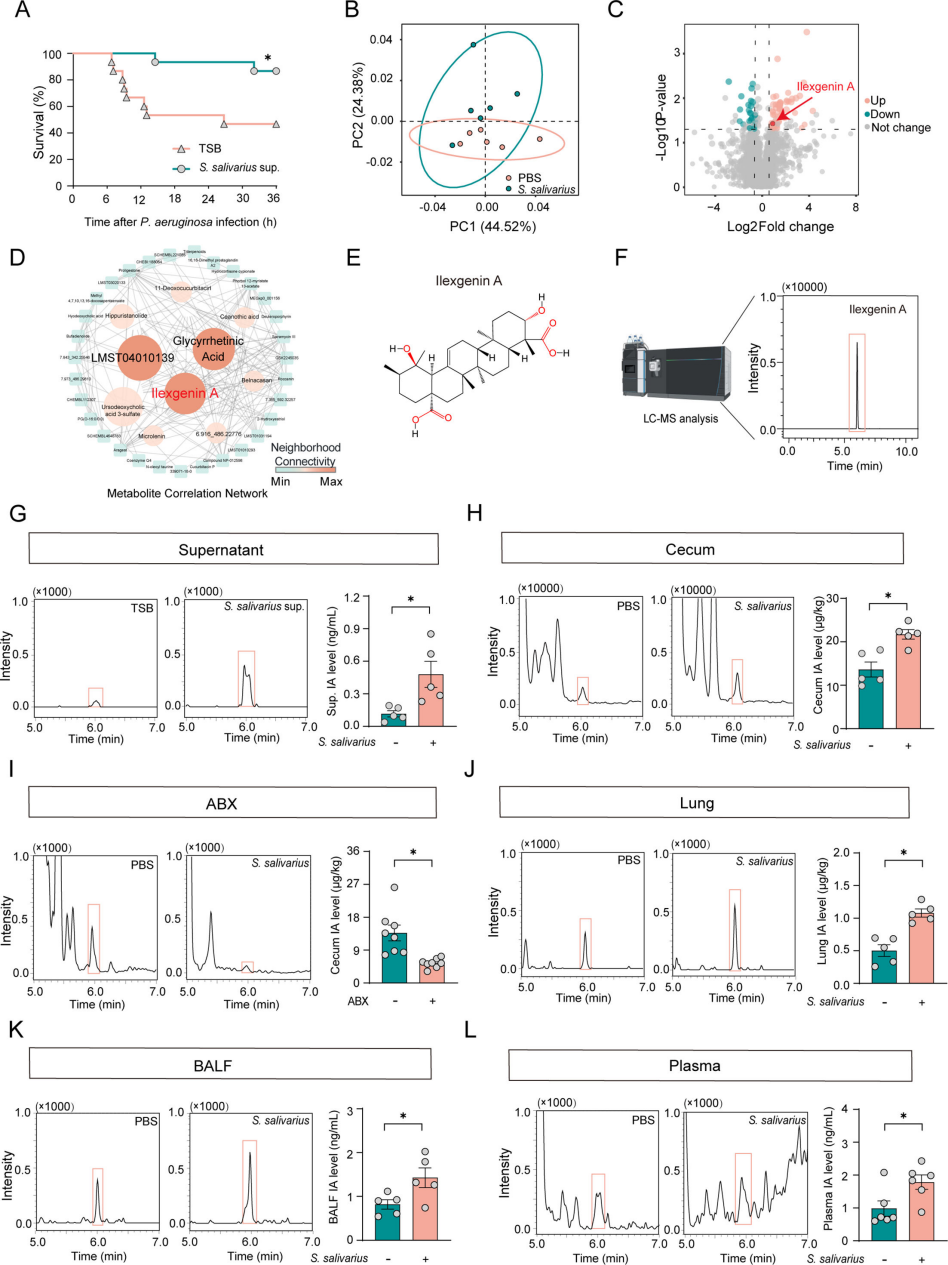
*S. salivarius* induces the biosynthesis of IA. (A) The survival curves of mice pretreated with 200 µL of TSB or *S. salivarius* culture supernatant (sup.) for 7 days after infection. *n* = 15. (B) Partial least squares discriminant analysis (PLS-DA) of metabolites in fecal samples from mice treated with PBS or *S. salivarius*. *n* = 6. (C) Volcano plots depicting differential metabolites among PBS or *S. salivarius*-treated mice. *n* = 6, *P* < 0.05, fold change ≥1.5 and variable importance in projection (VIP) ≥1.5 indicates significant differences. *n* = 6. (D) The metabolite correlation network reveals the interactions of the significant differential metabolites. *n* = 6. (E) The chemical structure of IA. (F) Chromatogram of IA standard obtained by LC–MS. (G) IA concentrations in culture sup. of *S. salivarius* by LC–MS. *n* = 5. (H) IA concentrations of cecum in mice with or without *S. salivarius* treatment were measured by LC–MS. *n* = 5. (I) IA concentrations of cecum in mice subjected to pretreatment with ABX. *n* = 8. (J–L) IA concentrations in the cecum, plasma, BALF, and lungs of mice with or without *S. salivarius* treatment following 7 days of oral administration. *n* = 5–6. All data are shown as mean ± SEM. Comparisons were assessed by two-tailed unpaired *t*-test. The survival rates of septic mice were analyzed using the Kaplan-Meier method with log-rank tests, *P < 0.05.

To validate this hypothesis, we first administered bacterial culture supernatants of *S. salivarius* to mice via gavage. As anticipated, these supernatants significantly reduced mortality rates in mice infected with *P. aeruginosa* (Fig. 3A). Subsequently, we performed a metabolomic analysis of the feces of mice gavaged with *S. salivarius*. Principal component analysis revealed distinct metabolic signatures in these subjects (Fig. 3B). Among these results, the volcano plot and metabolic interaction network highlight the important roles of LMST04010139, glycyrrhetinic acid, and IA (Fig. 3C and D). Given the reported anti-inflammatory effects of glycyrrhetinic acid and IA (17, 18), we analyzed changes in these two components in the supernatant of the *S. salivarius* culture medium. LC-MS analysis showed a significant increase in IA levels in the supernatant of the *S. salivarius* compared with blank TSB medium, whereas glycyrrhetinic acid did not (Fig. 3E through G and Fig. S2D and E). Subsequently, we further examined and found that compared with the control mice, the IA content in the cecum of mice in the *S. salivarius* group was significantly increased (Fig. 3H). Conversely, after antibiotic (ABX) treatment in mice, the levels of IA in the cecum were significantly reduced (Fig. 3I). Notably, the levels of IA were increased in the plasma, BALF, and lungs after the *S. salivarius* intervention (Fig. 3J through L).

In summary, these results indicate that *S. salivarius* may protect against pneumonia by metabolically deriving IA.

### IA treatment mitigates *P. aeruginosa*-induced pneumonia

**Fig 4:**
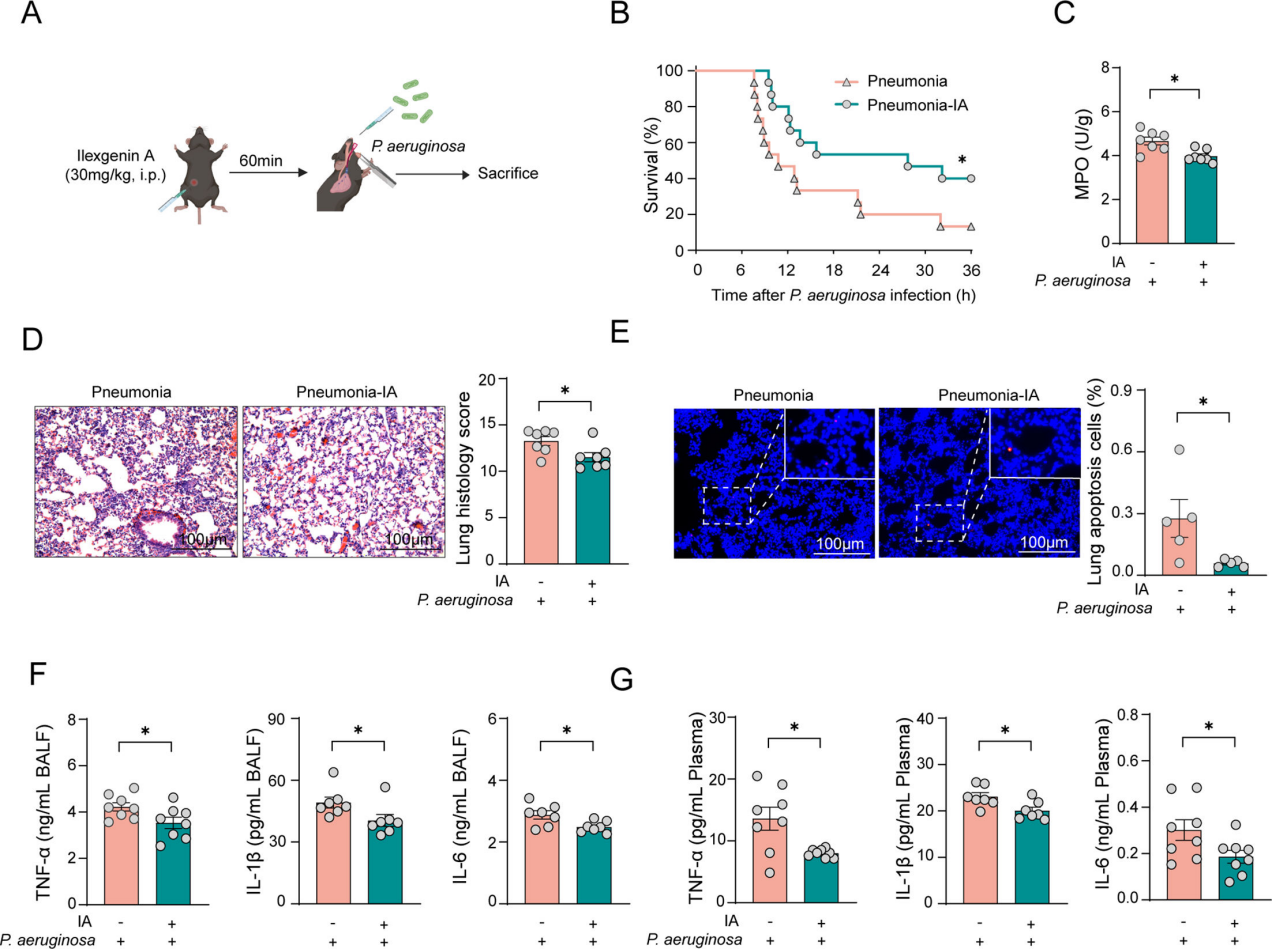
IA treatment mitigates *P. aeruginosa*-induced pneumonia. (A) Mice were intraperitoneally administered IA at a dose of 30 mg/kg 1 h prior to infection. (B) The survival curve of mice infected with *P. aeruginosa* under the intervention of IA. *n* = 15. (C) MPO activity in the lungs of *P. aeruginosa*-infected mice subjected to pretreatment with IA. *n* = 7. (D–E) H&E and TUNEL staining were performed to quantify lung injury and cell death percentages in IA-pretreated mice. *n* = 6. (F) Quantification of cytokines in the BALF from infected mice pretreated with IA. *n* = 6. (G) Quantification of cytokines in the plasma of infected mice pretreated with IA. *n* = 6. Scale bar, 100 µm. Data are shown as mean ± SEM. Comparisons were assessed by two-tailed unpaired *t*-test. The survival rates of septic mice were analyzed using the Kaplan-Meier method with log-rank tests, *P < 0.05.

To clarify whether IA can reduce the pathological severity of pneumonia caused by *P. aeruginosa*, we divided C57BL/6 mice into two groups: one group infected with *P. aeruginosa* and the other receiving an intraperitoneal injection of IA 1 h before infection (Fig. 4A). We then assessed the therapeutic effect of IA on the infection. Compared with the control group, the survival rate of the IA-treated mice significantly improved (Fig. 4B). Biochemical indicators showed that IA pretreatment reduced the infection-induced abnormal rise in lung MPO levels (Fig. 4C). Histopathological analysis indicated that alveolar wall thickening and inflammatory cell infiltration in lung tissue were alleviated in the IA treatment group compared with the control group (Fig. 4D). TUNEL results revealed that IA significantly reduced lung cell death due to infection (Fig. 4E). Additionally, the expression of inflammatory cytokines IL-6, IL-1β, and TNF-α in BALF was significantly reduced in the IA intervention group compared with the control group (Fig. 4F). Similarly, the increase in these inflammatory cytokines in the plasma was also alleviated in the IA intervention group (Fig. 4G). Finally, we evaluated the protective effect of IA on pneumonia damage induced by other bacterial infections (Fig. S3A and B). The results demonstrated that IA significantly improved the survival rates of mice infected with *K. pneumoniae* or *S. aureus*. In summary, our findings indicate that IA administration enhances the resistance of mice to bacterial infections.

### IA decreases inflammatory responses in macrophages

Subsequently, we investigated the potential mechanism by which IA exerts a protective effect against *P. aeruginosa*-induced pneumonia. We first determined whether IA affected bacterial counts. In fact, the total bacterial load in the lungs of mice with pneumonia was not affected by IA administration (Fig. S4A). Additionally, *P. aeruginosa* was cultured *in vitro* with varying concentrations of IA. Analysis of OD_600_ values revealed that the growth of *P. aeruginosa* was not affected compared with the control group (Fig. S4B). These findings indicate that IA does not influence the growth of *P. aeruginosa*.

**Fig 5:**
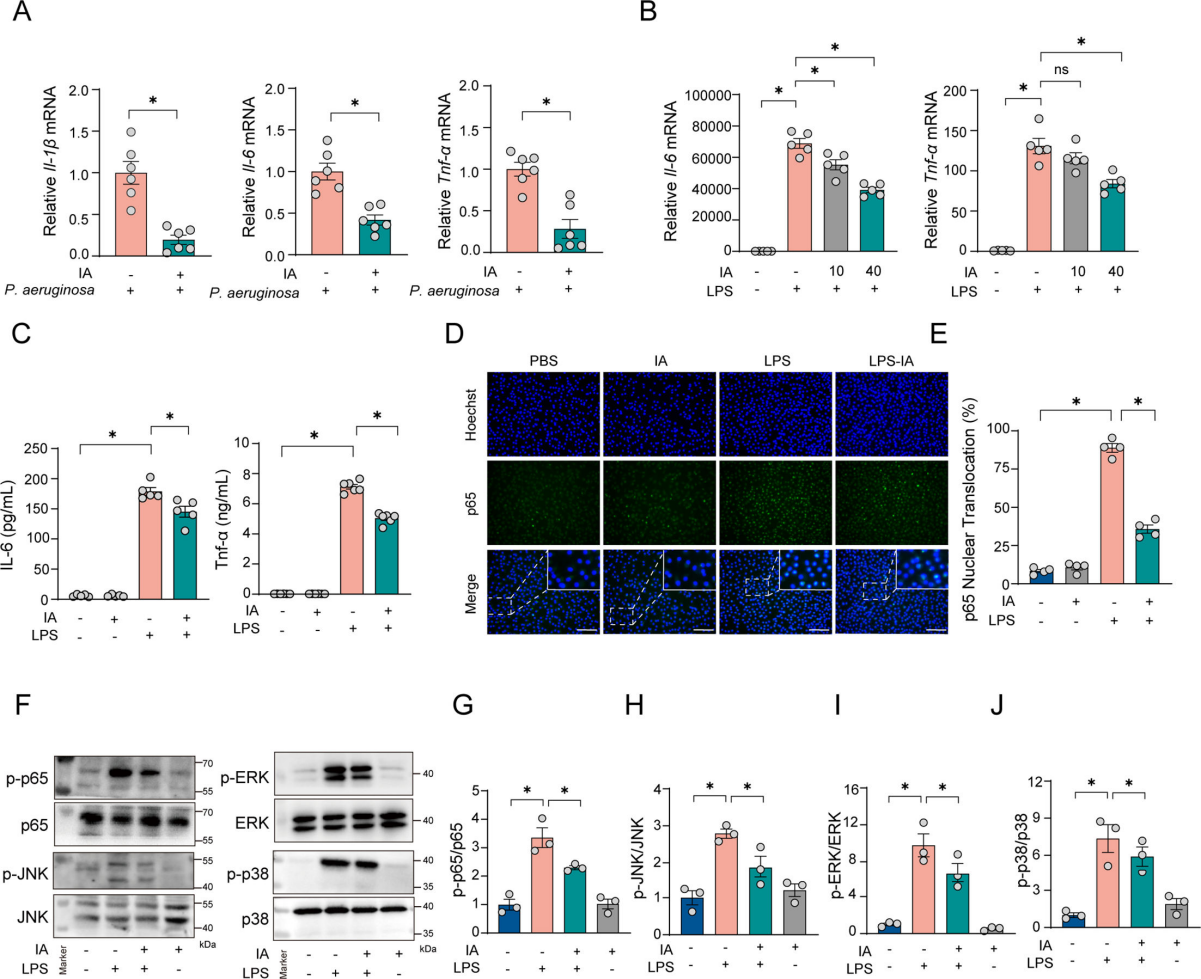
IA decreases inflammatory responses in macrophages. IA was administered 1 h before stimulation with LPS (100 ng/mL). Immunofluorescence was used to detect the nuclear translocation of p65 after 15 min of stimulation. Western blot was used to detect kinases at 30 min post-stimulation, and qRT-PCR and ELISA were used to detect mRNA and cytokines 6 h after stimulation. (A) The relative *IL-1*β, *IL-6,* and *TNF-*α mRNA levels in AMs from infected mice pretreated with IA *n* = 6. (B) The relative IL-6 and TNF-α mRNA levels were measured in BMDMs treated with or without IA by qRT-PCR. *n* = 5. (C) The concentrations of IL-6 and TNF-α were measured in BMDMs treated with or without IA by ELISA. *n* = 5–6. (D–E) p65 immunofluorescence staining quantifies the percentage of p65 translocation from the cytoplasm to the nucleus in BMDMs. *n* = 4. (F–J) The levels of p-p65, p-JNK, p-p38, and p-ERK in BMDMs with or without IA treatment. *n* = 3–4. Data are shown as mean ± SEM. Scale bar, 100 µm. ns: *P* > 0.05 was considered not statistically significant. *P < 0.05 was considered statistically significant. Comparisons were assessed using a one-way ANOVA analysis.

We next focused on the effects of IA on the host. The outcome of pneumonia largely depends on the host’s immune response, with macrophages being a major source of the excessive inflammation observed during the condition (19). Therefore, we aimed to investigate the effect of IA on inflammatory expression in macrophages. IA treatment significantly downregulated *Tnf-*α, *Il-6*, and *Il-1*β mRNA levels in alveolar macrophages (AMs) after *P. aeruginosa* infection compared with controls (Fig. 5A). Additionally, based on our *in vitro* experiments, we found that IA administration was able to diminish *Tnf-*α and *Il-6* gene expression in isolated bone marrow-derived macrophages (BMDMs) after lipopolysaccharide (LPS) challenge (Fig. 5B). As expected, enzyme-linked immunosorbent assay (ELISA) of cell culture supernatants indicated that LPS-stimulated BMDMs elevate levels of the inflammatory cytokines, whereas IA was able to reverse this increase (Fig. 5C). The NF-κB and MAPK signaling pathways, which function as critical upstream regulators of IL-6 and TNF-α, are essential in modulating cellular inflammatory responses (20). Subsequently, we investigated the impact of IA on NF-κB and MAPK pathways. For the NF-κB pathway, immunofluorescence observed that BMDMs underwent significant p65 nuclear translocation after LPS stimulation, substantially increasing the proportion of cells with positive nuclear translocation. In contrast, the IA intervention group showed a significant decrease in this proportion (Fig. 5D and E). Meanwhile, western blot results showed that LPS stimulation activated the phosphorylation level of p65 in BMDMs, which was reversed by IA (Fig. 5F and G). Additionally, regarding the effect on MAPK pathway proteins, the results showed that LPS stimulation increased the phosphorylation levels of ERK, JNK, and p38 in BMDMs, which were inhibited in the IA intervention group (Fig. 5H through J). As expected, a similar trend was observed in pneumonia-induced mice treated with IA. Following IA treatment, the phosphorylation levels of p65, JNK, ERK, and p38 in AMs were significantly reduced when compared with control mice infected with *P. aeruginosa* (Fig. S4C and D).

In summary, these results demonstrated that IA could inhibit the NF-κB and MAPK signaling pathways to mitigate the secretion of inflammatory cytokines in macrophages during proinflammatory stimulation.

### IA exerts anti-inflammatory effects by modulating the TLR4 pathway

**Fig 6:**
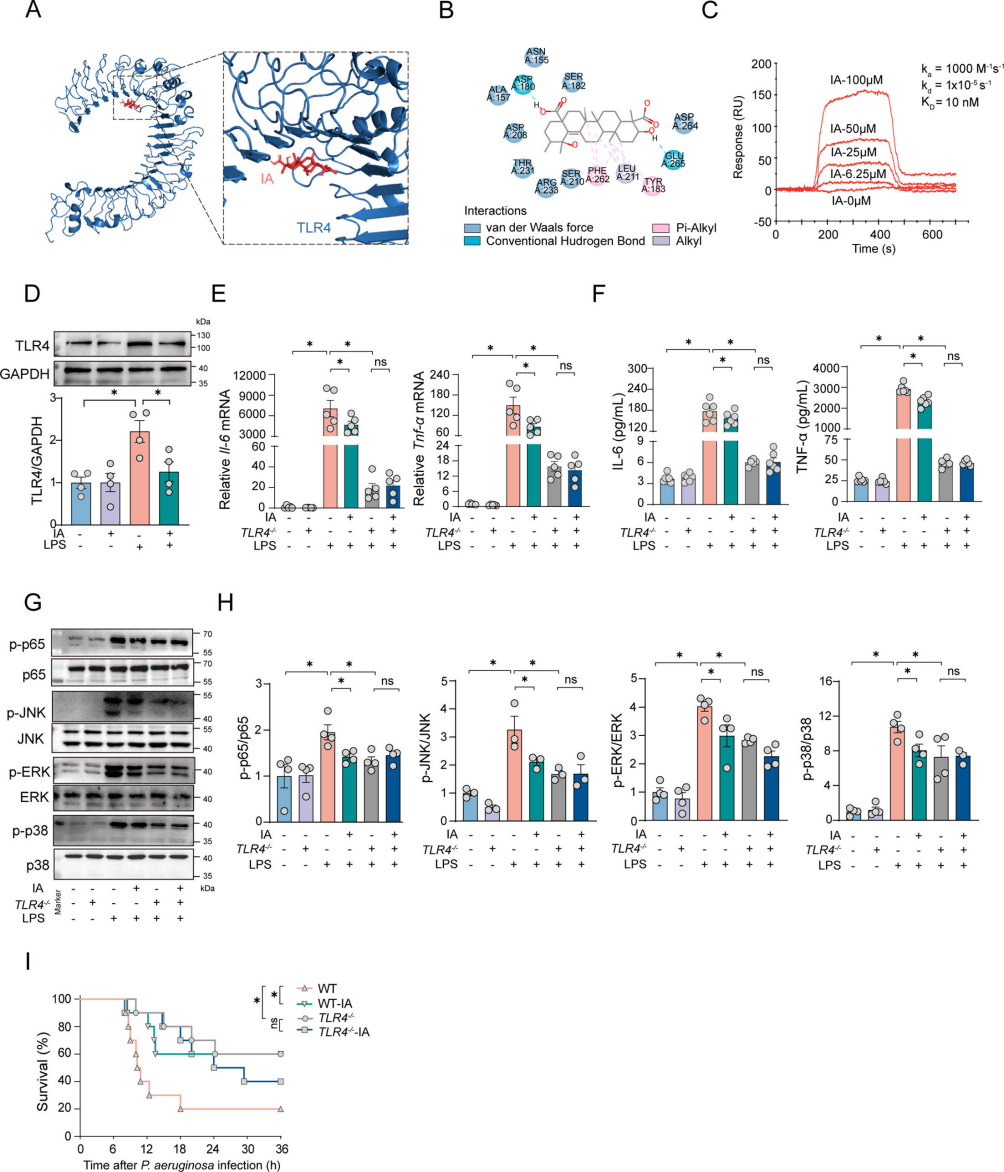
IA exerts anti-inflammatory effects by modulating the TLR4 pathway. (A, B) The molecular docking prediction results of TLR4 protein with IA were obtained through docking using AutoDock Vina. (C) SPR showed a direct interaction of IA with TLR4 protein. The TLR4 recombinant protein (2 mg) was immobilized on the SPRichip. The IA (dissolved in 5% DMSO) flowed at increasing concentrations. (D) The levels of TLR4 in BMDMs with or without IA treatment. *n* = 4. (E) Relative *IL-6* and *TNF-*α mRNA levels in BMDMs. IA was pretreated on WT-BMDMs and *TLR4^−/−^* BMDMs for 1 h, after which cells were harvested 6 h post LPS stimulation. *n* = 6. (F) Concentrations of IL-6 and TNF-α levels in BMDMs. *n* = 6. (G, H) The levels of p-p65, p-JNK, p-p38, and p-ERK in WT-BMDMs and *TLR4^−/−^* BMDMs with or without IA treatment. *n* = 3. (I) The survival curve of WT-mice and *TLR4*^−/−^-mice with or without IA treatment. *n* = 10. ns: *P* > 0.05 was considered not statistically significant. *P < 0.05 was considered statistically significant. Data were shown as mean ± SEM. Comparisons were assessed using a one-way ANOVA analysis.

TLR4 serves as an upstream receptor in the NF-κB and MAPK signaling pathways. Consequently, our objective was to investigate the role of TLR4 in mediating anti-inflammatory effects. Molecular docking was performed using AutoDock Vina. The results showed that IA can bind to TLR4 protein, with a binding energy of −7.6 kcal/mol, suggesting a theoretical autonomous docking ability (Fig. 6A and B). Furthermore, surface plasmon resonance (SPR) experiments confirmed the interaction between IA and TLR4 protein (Fig. 6C). Meanwhile, we observed that IA suppresses TLR4 expression in LPS-stimulated BMDMs (Fig. 6D).

Subsequently, we isolated *TLR4*^−/−^ BMDMs from *TLR4* knockout mice, whose genotype was confirmed via PCR-based analysis (Fig. S5A). qRT-PCR experiments indicated that the overexpression of pro-inflammatory cytokines induced by LPS was significantly reduced in *TLR4*^−/−^ BMDMs. Additionally, the therapeutic effect of IA was eliminated after *TLR4* knockout (Fig. 6E). The ELISA results showed a similar trend; after *TLR4* knockout, IA did not further reduce the levels of inflammatory factors IL-6 and TNF-α induced by LPS stimulation (Fig. 6F). Western blot experiments were used to detect protein phosphorylation levels in the NF-κB and MAPK signaling pathways. The results demonstrated that knocking out *TLR4* inhibited the LPS-induced phosphorylation of p65, p38, JNK, and ERK proteins. After knocking out the *TLR4* in BMDMs, IA did not show further inhibition of kinase phosphorylation levels (Fig. 6G and H). *In vivo*, we demonstrated that knockout of *TLR4* resulted in a reduced mortality rate in mice infected with *P. aeruginosa*. However, following *TLR4* knockout, the mortality rate in mice treated with IA did not show any further reduction (Fig. 6J). In summary, our results indicate that IA can bind to TLR4 protein and rely on it to inhibit excessive inflammatory expression in macrophages to alleviate pneumonia.

## DISCUSSION

Pneumonia in ICU patients involves a systemic immune response and alterations in organ function (21). Infection in distal organs potentially precipitates gut microbiota dysbiosis, thereby aggravating disease progression (22). In this study, we conducted a 16S rRNA sequencing analysis of the gut microbiota from non-pneumonia patients and pneumonia patients, revealing significant alterations in the microbial communities between the two groups, primarily characterized by shifts in microbial composition. Subsequently, we validated through FMT experiments that alterations in the gut microbiome could increase susceptibility to pneumonia. Mice colonized with fecal microbiota from patients with pneumonia exhibited exacerbated inflammatory responses and more pronounced pathological damage following *P. aeruginosa* infection. These findings suggest crosstalk between pneumonia and the gut microbiota in pneumonia patients, wherein the interaction between the microbiome and the lungs modulates the trajectory of pulmonary inflammation.

The gut microbiota can modulate immune changes in distant organs (23). Our previous studies identified *Lactobacillus vaginalis* and *Bifidobacterium adolescentis* within the gut as pivotal mediators of drug-induced hepatic injury in murine models (24, 25). However, the precise mechanisms by which the gut microbiome influences *P. aeruginosa*-induced pneumonia remain unclear. Here, we identified *S. salivarius* as a critical differential bacterium between pneumonia hosts and non-pneumonia individuals, suggesting that *S. salivarius* may be a key determinant of pneumonia susceptibility. *S. salivarius* is a naturally occurring commensal bacterium in humans, known for its beneficial role in mitigating and controlling various upper respiratory tract infections (26). However, its involvement in the pathogenesis of pneumonia remains poorly understood. In this study, using a *P. aeruginosa* infection mouse model, we demonstrated that gut colonization by *S. salivarius* ameliorates the inflammatory response, leading to improved infection outcomes. These findings underscore the critical role of *S. salivarius* in mediating gut microbiota-driven modulation of pulmonary inflammation. However, the mediating role of *S. salivarius* in alleviating pneumonia warrants further exploration.

It is widely known that the microbiome plays a crucial role in human health and disease by synthesizing various metabolites. For instance, short-chain fatty acids have been well-documented to provide protective effects against chronic obstructive pulmonary disease, asthma, and lung conditions related to COVID-19 (27–29). In this study, we identified a triterpenoid metabolite, ilexgenin A (IA), which is regulated by bacterial intervention. Metabolomic analysis revealed that *S. salivarius* modified the metabolic profile of the mice gut, with a notable increase in IA levels. Existing literature reports that some triterpenoids can be derived from gut microbiota metabolism to exert their biological effects (30–32). Therefore, it is reasonable to hypothesize a link between *S. salivarius* and IA. Subsequent LC-MS analysis confirmed that *S. salivarius* intervention significantly increased the concentration of IA in mice, with the highest levels observed in the cecum. This suggests that *S. salivarius* may metabolize IA in the intestine, which is then transported to lung tissue via the circulatory system. Multiple studies have shown that orally administered IA can be absorbed through the intestine into the bloodstream and exert protective effects on distal organs (33). For example, Yawen Lu et al. demonstrated that oral administration of IA in mice interfered with the maturation of sterol regulatory element-binding proteins in hepatocytes, thereby inhibiting fatty acid synthesis in the liver (34). Similarly, Bo Yang et al. reported that oral IA treatment improved myocardial ferroptosis and mitochondrial damage, thereby alleviating myocardial ischemia-reperfusion injury in mice (35). Taken together, these findings suggest that IA derived from the gut may serve as a key protective mechanism by which *S. salivarius* helps prevent the onset of pneumonia.

IA is a pentacyclic triterpenoid compound that has been experimentally validated to modulate lipid metabolism and inflammatory responses (36, 37). Recent studies on its anti-inflammatory properties have reported its ability to inhibit the activation of the NLRP3 inflammasome and reduce LPS-induced peritonitis in mice (38). To further explore whether the anti-inflammatory effects of IA extend to pulmonary inflammation, we administered IA to mice and observed a significant reduction in mortality rates and pulmonary damage resulting from *P. aeruginosa* infection. Given that macrophages are the primary source of excessive cytokine production during pneumonia, targeting these cells to mitigate the overproduction of inflammatory mediators is a logical therapeutic strategy (39). Here, we elucidate that IA effectively suppresses the expression of the inflammatory cytokines IL-6 and TNF-α in LPS-stimulated bone marrow-derived macrophages (BMDMs). In our study, exposure of macrophages to LPS resulted in the activation of both NF-κB and MAPK pathways. However, IA treatment significantly reduced NF-κB nuclear translocation and the phosphorylation of these kinases. Based on these findings, we conclude that IA exerts protective effects against *P. aeruginosa*-induced pneumonia by mitigating the excessive secretion of inflammatory cytokines from macrophages through the attenuation of NF-κB and MAPK pathway activation.

Finally, we sought to identify the molecular targets of IA to elucidate its regulatory mechanisms in inflammation. We identified TLR4 protein as the target of IA through molecular docking and SPR. TLR4 functions as a pivotal upstream target within the NF-κB and MAPK signaling pathways while also serving as the primary receptor for LPS on the cell membrane (40). In numerous pathological conditions, various antigens can activate TLR4, eliciting downstream inflammatory responses that exacerbate the host’s immune response, thereby aggravating the disease (41). Indeed, TLR4 antagonists have been reported to mitigate inflammatory diseases by suppressing TLR4 expression and reducing downstream inflammatory signaling pathways, thereby protecting against lung inflammation (42, 43). In this study, we identified IA as a ligand of TLR4 that reduces TLR4 overexpression and modulates downstream NF-κB and MAPK signaling pathways in a TLR4-dependent manner to produce anti-inflammatory effects.

Although the article combined clinical samples to explore the anti-pneumonia protective effects of *S. salivarius* and its metabolite IA, there are still certain limitations. First, the relatively small sample size for studying microbiota changes in pneumonia patients may limit the generalizability of the findings. Future studies will aim to include larger sample sizes to enhance the robustness and applicability of the conclusions. Moreover, although our functional data support an association between *S. salivarius* treatment and elevated IA levels *in vivo*, direct evidence for *de novo* bacterial synthesis of IA is lacking. IA may still be converted from its glycosidic form to the aglycone via biotransformation or other mechanisms (44, 45). Current evidence regarding biosynthetic intermediates, enzymes, or genetic pathways for IA biosynthesis remains insufficient, necessitating further investigation. Our previous study has demonstrated that intestinal bacterial β-galactosidases can biotransform dietary isoflavones, thereby influencing sepsis susceptibility (13). This highlights bacterial metabolic enzymes as a promising avenue for future research.

In conclusion, our research demonstrates that *S. salivarius* can induce the production of the metabolite IA, which subsequently binds to TLR4 and relies on it to inhibit downstream inflammatory cascades. This mechanism offers protection against pneumonia induced by *P. aeruginosa*. These findings provide new therapeutic strategies for pneumonia treatment from the perspective of the microbiota–immune axis.

## MATERIALS AND METHODS

### Mice

Eight-week-old male C57BL/6 mice were obtained from SPF Biotechnology Co., Ltd. (Beijing, China). Toll-like receptor 4 knockout (*TLR4*^–/–^) mice were sourced from GemPharmatech Co., Ltd. (Beijing, China). The mice were provided with unlimited access to food and water and kept in a temperature-regulated colony room under a 12 h light–dark cycle. All mouse experiments were conducted in accordance with protocols approved by the Institutional Animal Care and Use Committee of Southern Medical University, Guangzhou, China.

### Bacterial culture

*S. salivarius* (ATCC7073) was cultured in Tryptic Soy Broth (TSB) medium, whereas *P. aeruginosa* (ATCC27853), *K. pneumoniae* (ATCC10031), and *S. aureus* (ATCC43300) were cultured in Luria-Bertani medium. Subsequently, the strains were cultured overnight under 37°C aerobic conditions and finally aliquoted and stored in −80°C medium containing 20% glycerol for future use.

### Animal intervention

*P. aeruginosa*-pneumonia model was established as described previously (13). Briefly, mice were deeply anesthetized and intratracheally infected with *P. aeruginosa* (1.5 × 10^7^ CFU/mouse). Similarly, for pneumonia caused by other bacterial infections, the infection dose was 2 × 10⁸ CFU per mouse for *S. aureus* and 3 × 10⁸ CFU per mouse for *K. pneumoniae*. For the intervention of probiotics, mice were gavaged with *S. salivarius* (5 × 10^8^ CFU/mouse) once a day for 7 days. For IA (ChemFaces, Hubei, China) treatment, mice received IA (30 mg/kg) dissolved in sesame oil by intraperitoneal injection 60 min before *P. aeruginosa* infection. After modeling, mice were observed for 36 h survival or sacrificed at 6 h to collect tissues.

### Human samples

Fresh fecal samples from pneumonia and non-pneumonia patients were collected in the morning and immediately frozen at −80°C for preservation. The samples from pneumonia patients were collected from the ICU of the First People’s Hospital of Foshan. Detailed clinical information for registered pneumonia patients is listed in Table S1. Detailed clinical information for non-pneumonia patients is listed in Table S2.

### Fecal microbiota transplantation

For the FMT experiment (13), fecal samples were resuspended in PBS at a concentration of 0.125 g/mL. C57BL/6 mice were pretreated with a combination of ABX (100 mg/kg vancomycin, 200 mg/kg neomycin sulfate, 200 mg/kg metronidazole, and 200 mg/kg ampicillin) administered by oral gavage for 3 days to deplete the gut microbiota. Following this pretreatment, the mice received 200 µL of fecal suspension once daily for 5 consecutive days via oral gavage.

### Cell culture and therapy

For the cultivation of primary murine bone marrow-derived macrophages (BMDMs), the femurs and tibias were harvested from male C57BL/6 mice. The bone marrow was then flushed out with prechilled Dulbecco’s-modified Eagle’s medium (DMEM; Gibco, NY, USA). BMDMs were cultured in DMEM supplemented with 10% fetal bovine serum (Gibco, NY, USA), 1% penicillin-streptomycin (Gibco, NY, USA), and 20 ng/mL recombinant murine macrophage-colony-stimulating factor (M-CSF; Miltenyi Biotec, Gladbach, Germany). The cells were maintained in an incubator at 37°C with 5% CO2. The cells were pretreated with IA (40 µM) for 60 min before stimulation with LPS (Sigma, MO, USA) (100 ng/mL). Proteins were collected 30 min after stimulation for western blot analysis, and the cells and supernatants were collected 6 h later for qRT-PCR and ELISA.

### Isolation of alveolar macrophages

As previously described (19), AMs were isolated using anti-F4/80 microBeads (Miltenyi Biotec, Shanghai, China) according to the manufacturer’s instructions. Briefly, mouse BALF was filtered through a 40-µm nylon mesh and washed with MACS buffer. After cell counting, the suspension was incubated with anti-F4/80 microBeads on ice for 20 minutes. Labeled cells were passed through a MACS separator, and F4/80-positive cells were retained, washed, and eluted to obtain AMs.

### Microbial analysis

Fecal samples were gathered for DNA extraction using a commercial kit (Mabio, Guangzhou, China) following the manufacturer’s guidelines. The polymerase chain reaction (PCR) was conducted to amplify the variable region 4 (V4) of the bacterial 16S rRNA gene, employing primers V4F (5′-GTGTGYCAGCMGCCGCGGTAA-3′) and V4R (5′-CCGGACTACNVGGGTWTCTAAT-3′). Subsequently, DNA sequencing was carried out on an Illumina MiSeq PE250 platform. The raw sequencing data were analyzed with QIIME2 to profile the gut microbiota.

### Biochemical analysis

The cytokines TNF-α, IL-1β, and IL-6 in plasma, BALF, or cell culture supernatants were quantitated with a corresponding ELISA kit (Neobioscience, Shenzhen, China). MPO activity in the lungs was detected with commercial kits (Jiancheng Bioengineering Institute, Nanjing, China).

### Bacterial load assay

The bacterial load was determined as described previously (19). Briefly, lung tissues were collected from mice and subjected to DNA extraction using a universal DNA extraction kit (Mabio, Guangzhou, China). The extracted DNA, including both lung tissue and bacterial DNA, was analyzed by quantitative PCR (qPCR) targeting the 16S rRNA gene, which was normalized using 18S rRNA as an internal reference.

### Quantitative real-time PCR (qRT-PCR) analysis

RNA extraction was carried out using TRIzol reagent (Thermo Scientific, MA, USA), followed by reverse transcription with the ReverTra Ace qPCR RT Kit (Toyobo, Shanghai, China). qRT-PCR was conducted using a 7500 real-time PCR system (Applied Biosystems, CA, USA). A complete list of primers used for qRT-PCR can be found in Table S3.

### Western blot

For total protein extraction, cells were lysed on ice using RIPA buffer (Sigma, MO, USA), followed by heat denaturation in an SDS loading buffer for 5 min. The protein lysates were then separated by SDS-PAGE and transferred to a nitrocellulose membrane. Membranes were blocked with 5% bovine serum albumin (BSA) for 1 h at room temperature and then incubated overnight at 4°C with primary antibodies, including TLR4 (Cat# 14358S), p65 (Cat# 8242S), p-p65 (Cat# 3033S), JNK (Cat# 9252S), p-JNK (Cat# 9255S), ERK (Cat# 4695S), p-ERK (Cat# 9101S), p38 (Cat# 9212S), p-p38 (Cat# 9211S), and GAPDH (Cat# 2118S) (Cell Signaling Technology, MA, USA). The following day, the membranes were washed and incubated with secondary antibodies (Cell Signaling Technology, MA, USA) for 1 h at room temperature. Protein bands were visualized using an enhanced chemiluminescence assay (Biosharp, Beijing, China).

### Histopathological analysis

The lungs were harvested and fixed in 4% paraformaldehyde (Leagene, Beijing, China) for 36 h. The tissues were then embedded in paraffin, sectioned to a thickness of 5 µm, and stained with hematoxylin and eosin. A minimum of six fields per sample were randomly selected for microscopic observation. The severity of lung injury was scored 0–4 for each parameter, including alveolar congestion, hemorrhage, inflammatory cell infiltration, and thickness of the alveolar wall, with a maximum score of 16 (46).

TUNEL assay was conducted on paraffin-embedded tissue sections using a commercial kit (KeyGEN BioTECH, Nanjing, China) in accordance with the manufacturer’s instructions. Briefly, apoptotic cells were labeled with the TdT enzyme reaction solution and Streptavidin-TRITC labeling solution, whereas the cell nuclei were stained with DAPI. Apoptosis staining was observed under a fluorescence microscope with an excitation wavelength of 543 nm and an emission wavelength of 571 nm. For each slice, six fields of view were randomly captured, and the positive cells in the tissue were counted for biological statistics.

### Immunofluorescence

BMDMs were incubated with IA for 1 h, followed by stimulation with LPS for 15 min, then fixed with 4% PFA, permeabilized with 0.2% Triton X-100, and blocked with 5% BSA. After the reaction, the cells were incubated with an anti-p65 antibody overnight at 4°C and then with Alexa Fluor 488-conjugated anti-rabbit IgG (Abcam, MA, USA) for 1 h at room temperature. The immunofluorescent localization of p65 was observed using a fluorescence microscope. The proportion of nuclear translocation-positive cells was evaluated using LAS X software.

### Metabolomics analysis

Non-targeted metabolomic analysis was performed using a Vanquish UHPLC system coupled with an Orbitrap Q Exactive series mass spectrometer (Thermo Scientific, MA, USA). Sample preparation involved sonication in 90% (wt:vol) ultrapure water, followed by the addition of methanol and thorough vortexing. After centrifugation at 12,000 *× g* for 10 min at 4°C, the supernatants were collected. Subsequently, 100 µL of each sample was injected into a Hyperil Gold column (Thermo Scientific, MA, USA). In the positive polarity mode, the eluents used were eluent A (0.1% formic acid) and eluent B (methanol). The gradient elution procedure was as follows: 2% B, 1.5 min; 2%–100% B, 12.0 min; 100% B, 14.0 min; 100%–2% B, 14.1 min; and 2% B, 17 min. The flow rate was 200 µL/min. The source data were analyzed with Compound Discoverer 3.1 (Thermo Scientific, MA, USA). The volcano plot and PCA were generated using the online platform Bioinformatics (https://www.bioinformatics.com.cn). The metabolite correlation network was constructed using Cytoscape 3.8.0.

### Liquid chromatography–mass spectrometry (LC-MS) analysis

The bacterial culture supernatant was prepared by incubating Streptococcus salivarius in TSB medium for 24 h, followed by centrifugation at 6,000 *× g* for 5 min to remove the pellet and filtration through a 0.22-µm membrane to eliminate bacteria. Methanol was then used to extract the supernatant from the medium and tissue, followed by centrifugation at 16,000 × *g* for 15 min. The supernatant was dried using nitrogen gas and subsequently resuspended in methanol. Each sample (10 µL) was injected into an ACQUITY UPLC BEH C18 column (Waters, MA, USA). LC–MS analysis was performed with the Prelude SPLC system coupled with a TSQ Vantage triple quadrupole mass spectrometer (Thermo Scientific, MA, USA). For the detection of IA, the mobile phases used were 0.1% formic acid in water (A) and acetonitrile (B). The samples were eluted by gradients as follows: 10%–95% B, 7.0 min; 95% B, 9.0 min; and 10% B, 11.0 min. For the detection of glycyrrhetinic acid (ChemFaces, Hubei, China), the samples were eluted by gradients as follows: 10%–100% B, 2.0 min; 100% B, 3.5 min; and 10% B, 5.5 min. Data were processed with TraceFinderTM and Xcalibur software (Thermo Scientific, MA, USA).

### Molecular docking

The structure of TLR4 (PDB code: 2Z64) was obtained from the Protein Data Bank (https://www.rcsb.org). In addition, the 3D structure of IA (Compound CID: 21672638) was downloaded from the PubChem database (https://pubchem.ncbi.nlm.nih.gov/). Molecular modeling was performed in AutoDock Vina 1.2.5, followed by visualization in PyMOL 3.0.

### Mouse genotyping

Genotyping was performed following the method from The Jackson Laboratory (https://www.jax.org/strain/003752). Mouse tail tips (0.2–1 cm) were digested in lysis buffer at 55°C for 15 min and then inactivated at 95°C for 5 min, according to the instructions of the Mouse Tail DNA Extraction Kit (Beyotime, Shanghai, China). The extracted DNA was used as a template for PCR amplification, and the resulting products were analyzed by agarose gel electrophoresis. The primers of the *TLR4^+/+^* gene were F-5′-ATATGCATGATCAACACCACAG-3′ and R-5′-TTTCCATTGCTGCCCTATAG-3′ and primers the of *TLR4^−/−^* gene were F-5′-GCAAGTTTCTATATGCATTCTC-3′ and R-5′-CCTCCATTTCCAATAGGTAG-3′.

### Surface plasmon resonance

SPR analysis was conducted using the PlexArray HT A100 system (Plexera, USA). The protocol involved loading recombinant TLR4 protein (Nanjing Bioworld Biotech Co., Ltd., China) onto a 3D Dextran chip. The chip was then activated with equal amounts of 0.49 M 1-(3-Dimethylaminopropyl)−3-ethylcarbodiimide hydrochloride (EDC) and 0.1 M N-hydroxy-succinimide. Increasing concentrations of IA (dissolved in 5% DMSO) were flowed over the chip at 6.25, 12.5, 25, 50, and 100 µM, with a flow rate set at 2 µL/s. Data were collected using Plexera Data Explorer and analyzed with BIA evaluation software version 4.1.

### Quantification and statistical

Data were presented as mean ± standard error of the mean (SEM). Statistical analysis of data was performed by two-tailed unpaired *t*-test and one-way ANOVA analysis with GraphPad Prism 7 (GraphPad Software Inc., USA). ns: *P* > 0.05 was considered not statistically significant. *P < 0.05 was considered statistically significant. BioRender (http://biorender.com/) was used to create the flow charts.

## Data Availability

The 16s data have been deposited in the CNGB Sequence Archive (CNSA) of China National GeneBank DataBase (CNGBdb) with accession number CNP0006446.

## SUPPLEMENTAL MATERIAL

Supplemental Material msystems.00731-25-s0001.docx Figures S1 to S5; Tables S1 to S3.

ASM does not own the copyrights to Supplemental Material that may be linked to, or accessed through, an article. The authors have granted ASM a non-exclusive, world-wide license to publish the Supplemental Material files. Please contact the corresponding author directly for reuse.

## References

[1] de Benedictis FM, Kerem E, Chang AB, Colin AA, Zar HJ, Bush A. 2020. Complicated pneumonia in children. Lancet 396:786–798. doi:10.1016/S0140-6736(20)31550-6 32919518

[2] Mardian Y, Menur Naysilla A, Lokida D, Farida H, Aman AT, Karyana M, Lukman N, Kosasih H, Kline A, Lau C-Y. 2021. Approach to identifying causative pathogens of community-acquired pneumonia in children using culture, molecular, and serology tests. Front Pediatr 9:629318. doi:10.3389/fped.2021.629318 34123961 PMC8193353

[3] American Thoracic S, Infectious Diseases Society of A. 2005. Guidelines for the management, of adults with hospital-acquired, ventilator-associated, and healthcare-associated pneumonia. Am J Respir Crit Care Med 171:388–416. doi:10.1164/rccm.200405-644ST 15699079

[4] Micek ST, Wunderink RG, Kollef MH, Chen C, Rello J, Chastre J, Antonelli M, Welte T, Clair B, Ostermann H, Calbo E, Torres A, Menichetti F, Schramm GE, Menon V. 2015. An international multicenter retrospective study of Pseudomonas aeruginosa nosocomial pneumonia: impact of multidrug resistance. Crit Care 19:219. doi:10.1186/s13054-015-0926-5 25944081 PMC4446947

[5] Garnacho-Montero J, Ortiz-Leyba C, Fernández-Hinojosa E, Aldabó-Pallás T, Cayuela A, Marquez-Vácaro JA, Garcia-Curiel A, Jiménez-Jiménez FJ. 2005. Acinetobacter baumannii ventilator-associated pneumonia: epidemiological and clinical findings. Intensive Care Med 31:649–655. doi:10.1007/s00134-005-2598-0 15785929

[6] Torres A, Niederman MS, Chastre J, Ewig S, Fernandez-Vandellos P, Hanberger H, Kollef M, Li Bassi G, Luna CM, Martin-Loeches I, Paiva JA, Read RC, Rigau D, Timsit JF, Welte T, Wunderink R. 2017. International ERS/ESICM/ESCMID/ALAT guidelines for the management of hospital-acquired pneumonia and ventilator-associated pneumonia. Eur Respir J 50:1700582. doi:10.1183/13993003.00582-2017 28890434

[7] Berra L, Sampson J, Wiener-Kronish J. 2010. Pseudomonas aeruginosa: acute lung injury or ventilator-associated pneumonia? Minerva Anestesiol 76:824–832. 20935618

[8] Fangous M-S, Gosset P, Galakhoff N, Gouriou S, Guilloux C-A, Payan C, Vallet S, Héry-Arnaud G, Le Berre R. 2021. Priming with intranasal lactobacilli prevents Pseudomonas aeruginosa acute pneumonia in mice. BMC Microbiol 21:195. doi:10.1186/s12866-021-02254-7 34182930 PMC8237558

[9] Cho NA, Strayer K, Dobson B, McDonald B. 2024. Pathogenesis and therapeutic opportunities of gut microbiome dysbiosis in critical illness. Gut Microbes 16:2351478. doi:10.1080/19490976.2024.2351478 38780485 PMC11123462

[10] Le Guern R, Grandjean T, Stabler S, Bauduin M, Gosset P, Kipnis É, Dessein R. 2023. Gut colonisation with multidrug-resistant Klebsiella pneumoniae worsens Pseudomonas aeruginosa lung infection. Nat Commun 14:78. doi:10.1038/s41467-022-35767-4 36604442 PMC9816093

[11] Tang YX, Chen LQ, Yang J, Zhang SQ, Jin J, Wei Y. 2024. Gut microbes improve prognosis of Klebsiella pneumoniae pulmonary infection through the lung-gut axis. Front Cell Infect Microbiol 14. doi:10.3389/fcimb.2024.1392376 PMC11188585 38903943

[12] Nagata N, Takeuchi T, Masuoka H, Aoki R, Ishikane M, Iwamoto N, Sugiyama M, Suda W, Nakanishi Y, Terada-Hirashima J, et al.. 2023. Human gut microbiota and its metabolites impact immune responses in COVID-19 and its complications. Gastroenterology 164:272–288. doi:10.1053/j.gastro.2022.09.024 36155191 PMC9499989

[13] Chen X, Wu R, Li L, Zeng Y, Chen J, Wei M, Feng Y, Chen G, Wang Y, Lin L, Luo H, Chen A, Zeng Z, He F, Bai Y, Zhang S, Han Y, Wang Z, Zhao X, Xiao W, Jiang Y, Gong S. 2023. Pregnancy-induced changes to the gut microbiota drive macrophage pyroptosis and exacerbate septic inflammation. Immunity 56:336–352. doi:10.1016/j.immuni.2023.01.015 36792573

[14] Rastogi S, Mohanty S, Sharma S, Tripathi P. 2022. Possible role of gut microbes and host’s immune response in gut-lung homeostasis. Front Immunol 13:954339. doi:10.3389/fimmu.2022.954339 36275735 PMC9581402

[15] Zhang F, Lau RI, Liu Q, Su Q, Chan FKL, Ng SC. 2023. Gut microbiota in COVID-19: key microbial changes, potential mechanisms and clinical applications. Nat Rev Gastroenterol Hepatol 20:323–337. doi:10.1038/s41575-022-00698-4 36271144 PMC9589856

[16] Budden KF, Gellatly SL, Wood DLA, Cooper MA, Morrison M, Hugenholtz P, Hansbro PM. 2017. Emerging pathogenic links between microbiota and the gut-lung axis. Nat Rev Microbiol 15:55–63. doi:10.1038/nrmicro.2016.142 27694885

[17] Chen BD, Zhu DC, Xie CL, Shi YF, Ni LB, Zhang HW, Li SL, Lu JJ, Xiao J, Xia WY, Huang CG, Wang XY. 2021. 18β-Glycyrrhetinic acid inhibits IL-1β-induced inflammatory response in mouse chondrocytes and prevents osteoarthritic progression by activating Nrf2. Food Funct 12:8399–8410. doi:10.1039/D1FO01379C 34369548

[18] Li Y, Yang J, Chen MH, Wang Q, Qin MJ, Zhang T, Chen XQ, Liu BL, Wen XD. 2015. Ilexgenin A inhibits endoplasmic reticulum stress and ameliorates endothelial dysfunction via suppression of TXNIP/NLRP3 inflammasome activation in an AMPK dependent manner. Pharmacol Res 99:101–115. doi:10.1016/j.phrs.2015.05.012 26054569

[19] Gong S, Yan Z, Liu Z, Niu M, Fang H, Li N, Huang C, Li L, Chen G, Luo H, Chen X, Zhou H, Hu J, Yang W, Huang Q, Schnabl B, Chang P, Billiar TR, Jiang Y, Chen P. 2019. Intestinal Microbiota mediates the susceptibility to polymicrobial sepsis-induced liver injury by granisetron generation in mice. Hepatology:1751–1767. doi:10.1002/hep.30361 30506577

[20] Zhao WM, Ma L, Cai C, Gong XH. 2019. Caffeine inhibits NLRP3 inflammasome activation by suppressing MAPK/NF-κB and A2aR signaling in LPS-induced THP-1 macrophages. Int J Biol Sci 15:1571–1581. doi:10.7150/ijbs.34211 31360100 PMC6643212

[21] Blot S, Ruppé E, Harbarth S, Asehnoune K, Poulakou G, Luyt C-E, Rello J, Klompas M, Depuydt P, Eckmann C, Martin-Loeches I, Povoa P, Bouadma L, Timsit J-F, Zahar J-R. 2022. Healthcare-associated infections in adult intensive care unit patients: changes in epidemiology, diagnosis, prevention and contributions of new technologies. Intensi Crit Care Nursing 70:103227. doi:10.1016/j.iccn.2022.103227 PMC8892223 35249794

[22] Gebrayel P, Nicco C, Al Khodor S, Bilinski J, Caselli E, Comelli EM, Egert M, Giaroni C, Karpinski TM, Loniewski I, Mulak A, Reygner J, Samczuk P, Serino M, Sikora M, Terranegra A, Ufnal M, Villeger R, Pichon C, Konturek P, Edeas M. 2022. Microbiota medicine: towards clinical revolution. J Transl Med 20:111. doi:10.1186/s12967-022-03296-9 35255932 PMC8900094

[23] Campbell C, Kandalgaonkar MR, Golonka RM, Yeoh BS, Vijay-Kumar M, Saha P. 2023. Crosstalk between gut microbiota and host immunity: impact on inflammation and immunotherapy. Biomedicines 11:294. doi:10.3390/biomedicines11020294 36830830 PMC9953403

[24] Zeng Y, Wu R, Wang F, Li S, Li L, Li Y, Qin P, Wei M, Yang J, Wu J, Chen A, Ke G, Yan Z, Yang H, Chen Z, Wang Z, Xiao W, Jiang Y, Chen X, Zeng Z, Zhao X, Chen P, Gong S. 2023. Liberation of daidzein by gut microbial β-galactosidase suppresses acetaminophen-induced hepatotoxicity in mice. Cell Host Microbe 31:766–780. doi:10.1016/j.chom.2023.04.002 37100057

[25] Qin P, Li Y, Su Y, Wang Z, Wu R, Liang X, Zeng Y, Guo P, Yu Z, Huang X, Yang H, Zeng Z, Zhao X, Gong S, Han J, Chen Z, Xiao W, Chen A. 2024. Bifidobacterium adolescentis-derived hypaphorine alleviates acetaminophen hepatotoxicity by promoting hepatic Cry1 expression. J Transl Med 22:525. doi:10.1186/s12967-024-05312-6 38822329 PMC11143572

[26] Burton JP, Cowley S, Simon RR, McKinney J, Wescombe PA, Tagg JR. 2011. Evaluation of safety and human tolerance of the oral probiotic Streptococcus salivarius K12: a randomized, placebo-controlled, double-blind study. Food Chem Toxicol 49:2356–2364. doi:10.1016/j.fct.2011.06.038 21722694

[27] Kotlyarov S. 2022. Role of short-chain fatty acids produced by gut microbiota in innate lung immunity and pathogenesis of the heterogeneous course of chronic obstructive pulmonary disease. Int J Mol Sci 23:4768. doi:10.3390/ijms23094768 35563159 PMC9099629

[28] Kotlyarov S, Kotlyarova A. 2021. Anti-inflammatory function of fatty acids and involvement of their metabolites in the resolution of inflammation in chronic obstructive pulmonary disease. Int J Mol Sci 22:12803. doi:10.3390/ijms222312803 34884621 PMC8657960

[29] Yuan G, Wen S, Zhong X, Yang X, Xie L, Wu X, Li X. 2023. Inulin alleviates offspring asthma by altering maternal intestinal microbiome composition to increase short-chain fatty acids. PLoS One 18:e0283105. doi:10.1371/journal.pone.0283105 37014871 PMC10072493

[30] Tang X, Zeng T, Deng W, Zhao W, Liu Y, Huang Q, Deng Y, Xie W, Huang W. 2025. Gut microbe-derived betulinic acid alleviates sepsis-induced acute liver injury by inhibiting macrophage NLRP3 inflammasome in mice. mBio 16:e03020-24. doi:10.1128/mbio.03020-24 39887250 PMC11898617

[31] Liu S, Zhang Z, Wang X, Ma Y, Ruan H, Wu X, Li B, Mou X, Chen T, Lu Z, Zhao W. 2024. Biosynthetic potential of the gut microbiome in longevous populations. Gut Microbes 16:2426623. doi:10.1080/19490976.2024.2426623 39529240 PMC11559365

[32] Yao L, Wang J, He J, Huang L, Gao W. 2021. Endophytes, biotransforming microorganisms, and engineering microbial factories for triterpenoid saponins production. Crit Rev Biotechnol 41:249–272. doi:10.1080/07388551.2020.1869691 33472430

[33] Kuang GJ, Yi H, Zhu MJ, Zhou J, Shang XY, Zhao ZX, Zhu CC, Liao QF, Guan SX, Zhang L. 2017. Study of absorption characteristics of the total saponins from radix Ilicis pubescentis in an in situ single-pass intestinal perfusion (SPIP) rat model by using ultra performance liquid chromatography (UPLC). Molecules 22:1867. doi:10.3390/molecules22111867 29104273 PMC6150237

[34] Lu YW, Ma JJ, Li P, Liu BL, Wen XD, Yang J. 2022. Ilexgenin A restrains CRTC2 in the cytoplasm to prevent SREBP1 maturation via AMP kinase activation in the liver. Br J Pharmacol 179:958–978. doi:10.1111/bph.15369 33434948

[35] Yang B, Jue XY, Luo SF, Tan ZB, Yang LN, Feng YT, Tan YZ, Liu B, Zhang JZ, Deng B, Wu WW, Zhang SW. 2025. Ilexgenin A alleviates myocardial ferroptosis in response to ischemia reperfusion injury via the SIRT1 pathway. Phytother Res 39:938–956. doi:10.1002/ptr.8414 39698933

[36] Sun W, Liu C, Zhang Y, Qiu X, Zhang L, Zhao H, Rong Y, Sun Y. 2017. Ilexgenin A, a novel pentacyclic triterpenoid extracted from aquifoliaceae shows reduction of LPS-induced peritonitis in mice. Eur J Pharmacol 797:94–105. doi:10.1016/j.ejphar.2017.01.019 28104349

[37] Patil R, Chikhale R, Khanal P, Gurav N, Ayyanar M, Sinha S, Prasad S, Dey YN, Wanjari M, Gurav SS. 2021. Computational and network pharmacology analysis of bioflavonoids as possible natural antiviral compounds in COVID-19. Inform Med Unlocked 22:100504. doi:10.1016/j.imu.2020.100504 33363251 PMC7756171

[38] Shao BZ, Xu ZQ, Han BZ, Su DF, Liu C. 2015. NLRP3 inflammasome and its inhibitors: a review. Front Pharmacol 6:262. doi:10.3389/fphar.2015.00262 26594174 PMC4633676

[39] Dukhinova M, Kokinos E, Kuchur P, Komissarov A, Shtro A. 2021. Macrophage-derived cytokines in pneumonia: linking cellular immunology and genetics. Cytokine Growth Factor Rev 59:46–61. doi:10.1016/j.cytogfr.2020.11.003 33342718 PMC8035975

[40] Ciesielska A, Matyjek M, Kwiatkowska K. 2021. TLR4 and CD14 trafficking and its influence on LPS-induced pro-inflammatory signaling. Cell Mol Life Sci 78:1233–1261. doi:10.1007/s00018-020-03656-y 33057840 PMC7904555

[41] Root-Bernstein R. 2020. Synergistic activation of toll-like and NOD receptors by complementary antigens as facilitators of autoimmune disease: review, model and novel predictions. Int J Mol Sci 21:4645. doi:10.3390/ijms21134645 32629865 PMC7369971

[42] Tang R, Zhang J, Zhang R, Li X, Lv R, Nan H, Liu J, Zhao Z, He W, Wang L. 2023. Huoxiang zhengqi oral liquid attenuates LPS‐induced acute lung injury by modulating short‐chain fatty acid levels and TLR4/NF‐ κ B p65 pathway. Biomed Res Int 2023:6183551. doi:10.1155/2023/6183551 36845637 PMC9957650

[43] Guo Y, Zhang H, Lv Z, Du Y, Li D, Fang H, You J, Yu L, Li R. 2023. Up-regulated CD38 by daphnetin alleviates lipopolysaccharide-induced lung injury via inhibiting MAPK/NF-κB/NLRP3 pathway. Cell Commun Signal 21:66. doi:10.1186/s12964-023-01041-3 36998049 PMC10061746

[44] Chen X-Q, Zan K, Yang J, Liu X-X, Mao Q, Zhang L, Lai M-X, Wang Q. 2011. Quantitative analysis of triterpenoids in different parts of Ilex hainanensis, Ilex stewardii and Ilex pubescens using HPLC–ELSD and HPLC–MSn and antibacterial activity. Food Chem 126:1454–1459. doi:10.1016/j.foodchem.2010.11.136

[45] Shen Y, Gao Y, Yang G, Zhao Z, Zhao Y, Gao L, Zhao L, Li S. 2023. Transformation of ginsenosides by Lactiplantibacillus plantarum MB11 fermentation: minor ginsenosides conversion and enhancement of anti-colorectal cancer activity. Molecules 29:27. doi:10.3390/molecules29010027 38202610 PMC10780060

[46] Kubiak BD, Albert SP, Gatto LA, Snyder KP, Maier KG, Vieau CJ, Roy S, Nieman GF. 2010. Peritoneal negative pressure therapy prevents multiple organ injury in a chronic porcine sepsis and ischemia/reperfusion model. Shock 34:525–534. doi:10.1097/SHK.0b013e3181e14cd2 20823698

