# Supplementary material for: Streptococcus salivarius-derived ilexgenin A alleviates pneumonia through the gut-lung axis
Source: mSystems. 2025 Jul 30;10(8):e00731-25. doi: 10.1128/msystems.00731-25 (PMC12363194; doi:10.1128/msystems.00731-25)
Supplement: Supplemental Material — Figures S1 to S5; Tables S1 to S3. [file msystems.00731-25-s0001.docx]

**Supplemental information**


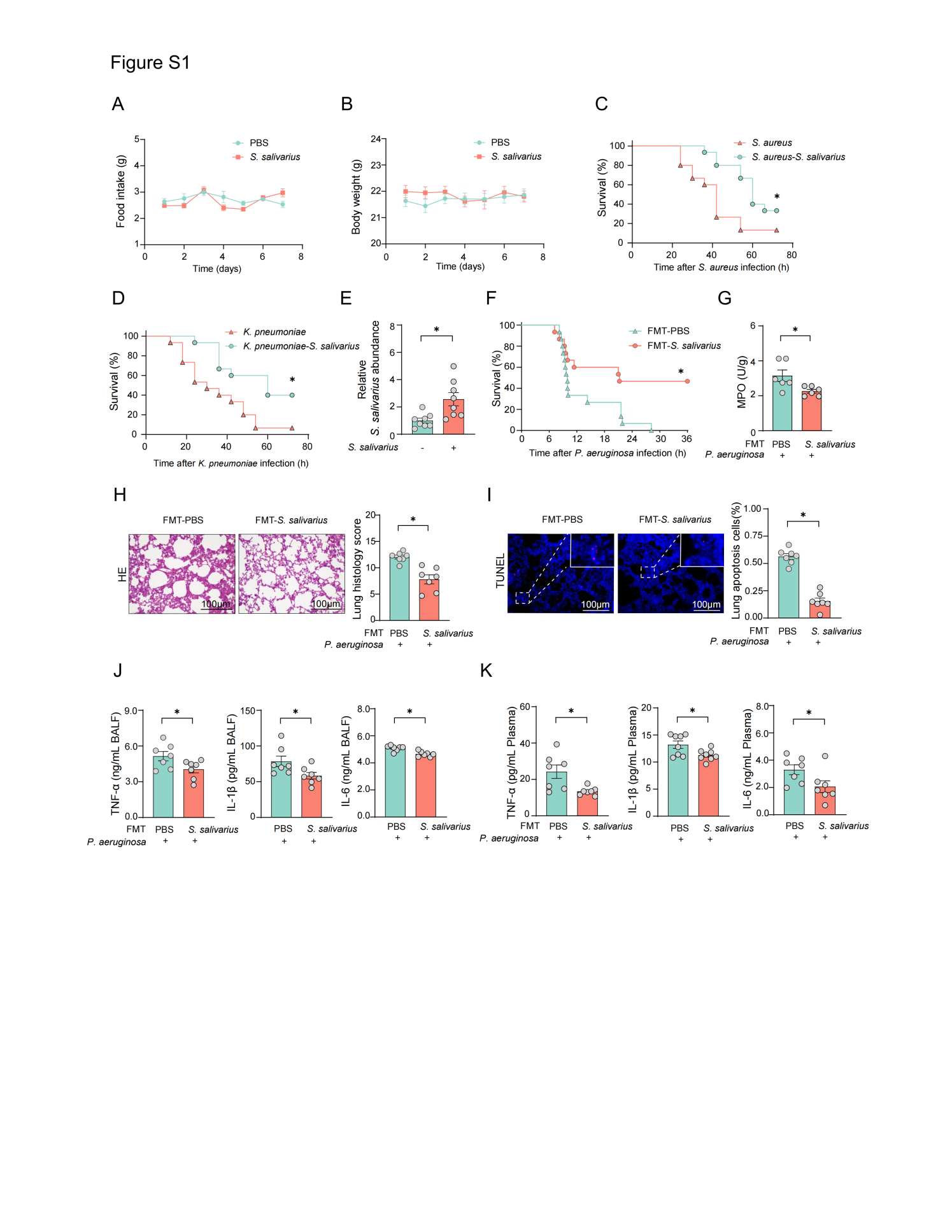


**Figure S1. Intestinal colonization by *S. salivarius* alleviates pneumonia.** **A, B** Body weight and food intake were measured daily in mice with or without *S. salivarius* (5×10^8^ CFU per mouse) treatment for 7 days. n=9. **C** Survival curve of mice pre-treated with *S. salivarius* against *S. aureus* infection over 72 hours. n=15. **D** Survival curve of mice pre-treated with *S. salivarius* against *K. pneumoniae* infection over 72 hours. n=15. **E** Relative abundance of *S. salivarius* in mice gavaged with *S. salivarius*. n=8. **F** The survival curve of mice received fecal suspension from mice gavaged with *S. salivarius*. n=15. **G** Effect of pretreatment with fecal suspension from mice gavaged with *S. salivarius* on MPO in lung from septic mice at 6 h after *P. aeruginosa* infection. **H** Histopathological evaluation of lung from pneumonia. n=6. Mice subjected to preadministration of fecal suspension from mice gavaged with or without *S. salivarius*. n=7. **I** TUNEL staining of lung from pneumonia mice received fecal suspension from mice gavaged with or without *S. salivarius* and quantification of dead cells. n=7. **J, K** Effect of pretreatment with fecal suspension from mice gavaged with or without *S. salivarius* on cytokines in BALF and plasma of pneumonia mice. n=7-8. Scale bar, 100 µm. Data are shown as mean ± SEM. Comparisons were assessed by a two-tailed unpaired Student's t-test. **p*<0.05.


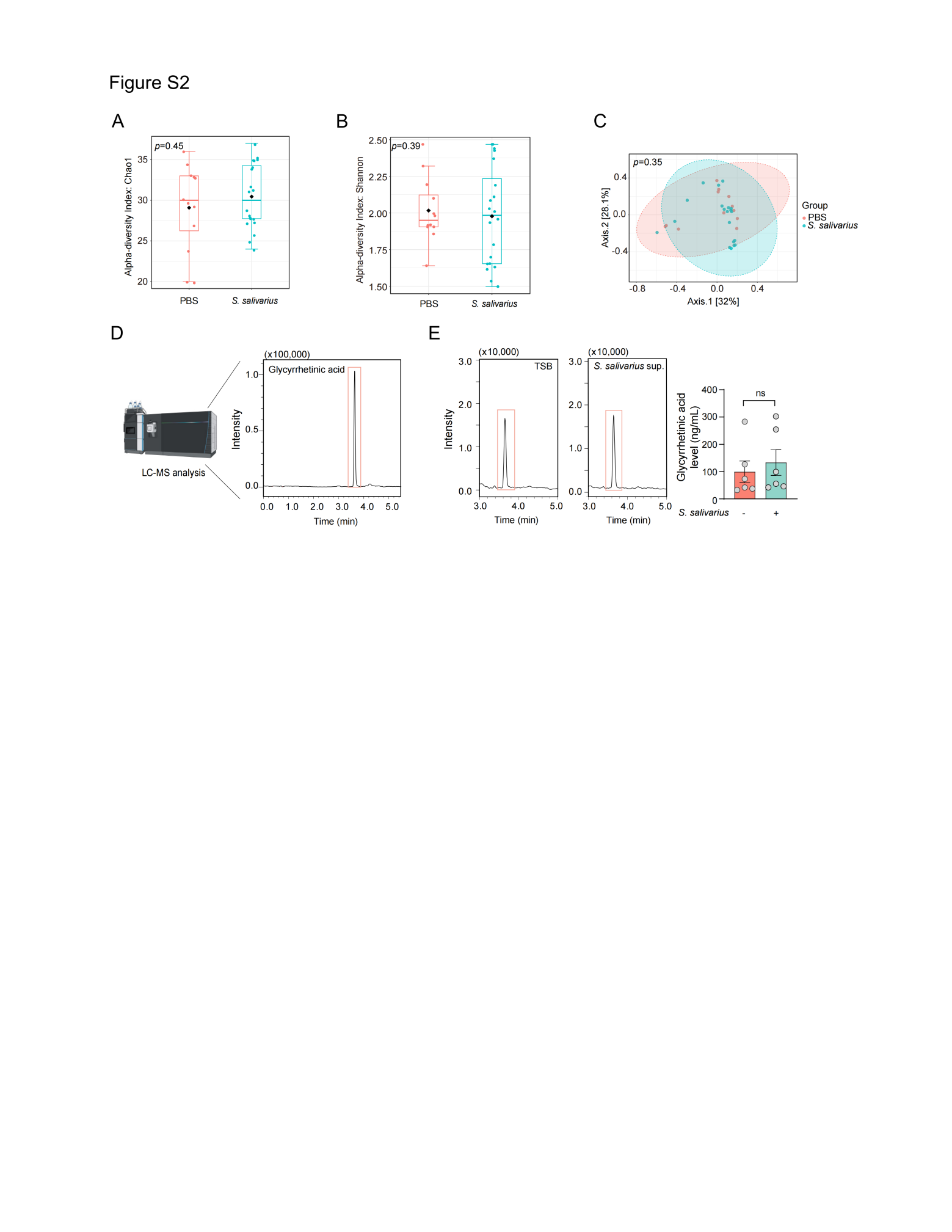


**Figure S2. Effects of oral administration of *S. salivarius* on gut microbiota and metabolites in mice. A** Chao1 diversity index of fecal samples collected from mice orally administered with *S. salivarius*. n=12-20. **B** Shannon’s diversity index of fecal samples collected from mice orally administered with *S. salivarius*. n=12-20. **C** The principal coordinates analysis (PCoA) of the gut microbiota in mice treated with or without *S. salivarius* based on Bray–Curtis distance. n=12-20. **D** Chromatogram of glycyrrhetinic acid standard obtained by LC–MS. **E** Glycyrrhetinic acid concentrations in culture supernatants (sup.) of *S. salivarius* by LC–MS. n=6. Data are shown as mean ± SEM. **p*<0.05 were assessed by a two-tailed unpaired Student's t-test.


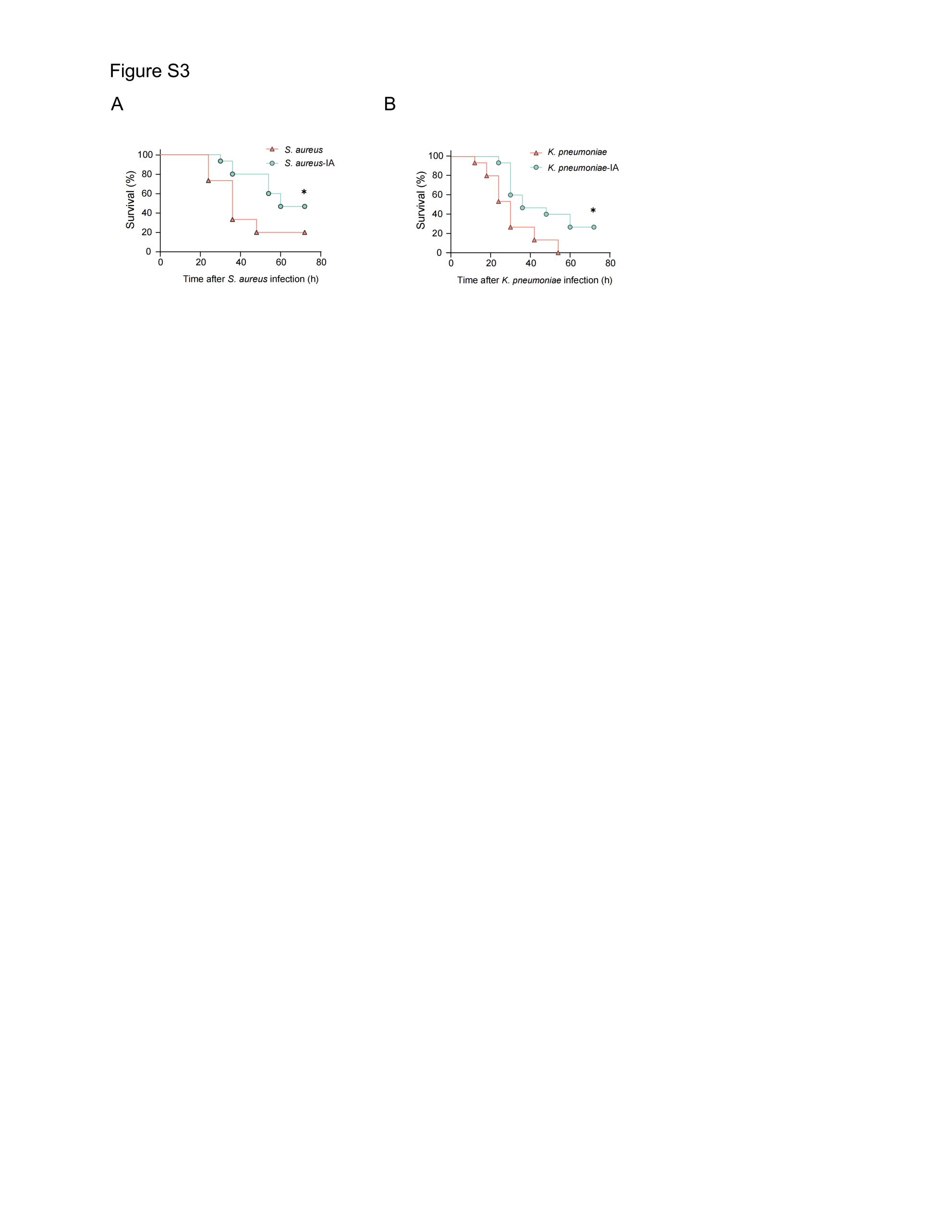


**Figure S3. The protective effect of IA on bacterial infectious pneumonia. A** Survival curve of mice pre-treated with IA against *S. aureus* infection over 72 hours. n=15. **D** Survival curve of mice pre-treated with IA against *K. pneumoniae* infection over 72 hours. n=15. The survival rates of septic mice were analyzed using Kaplan-Meier method with log-rank test, **p*<0.05.


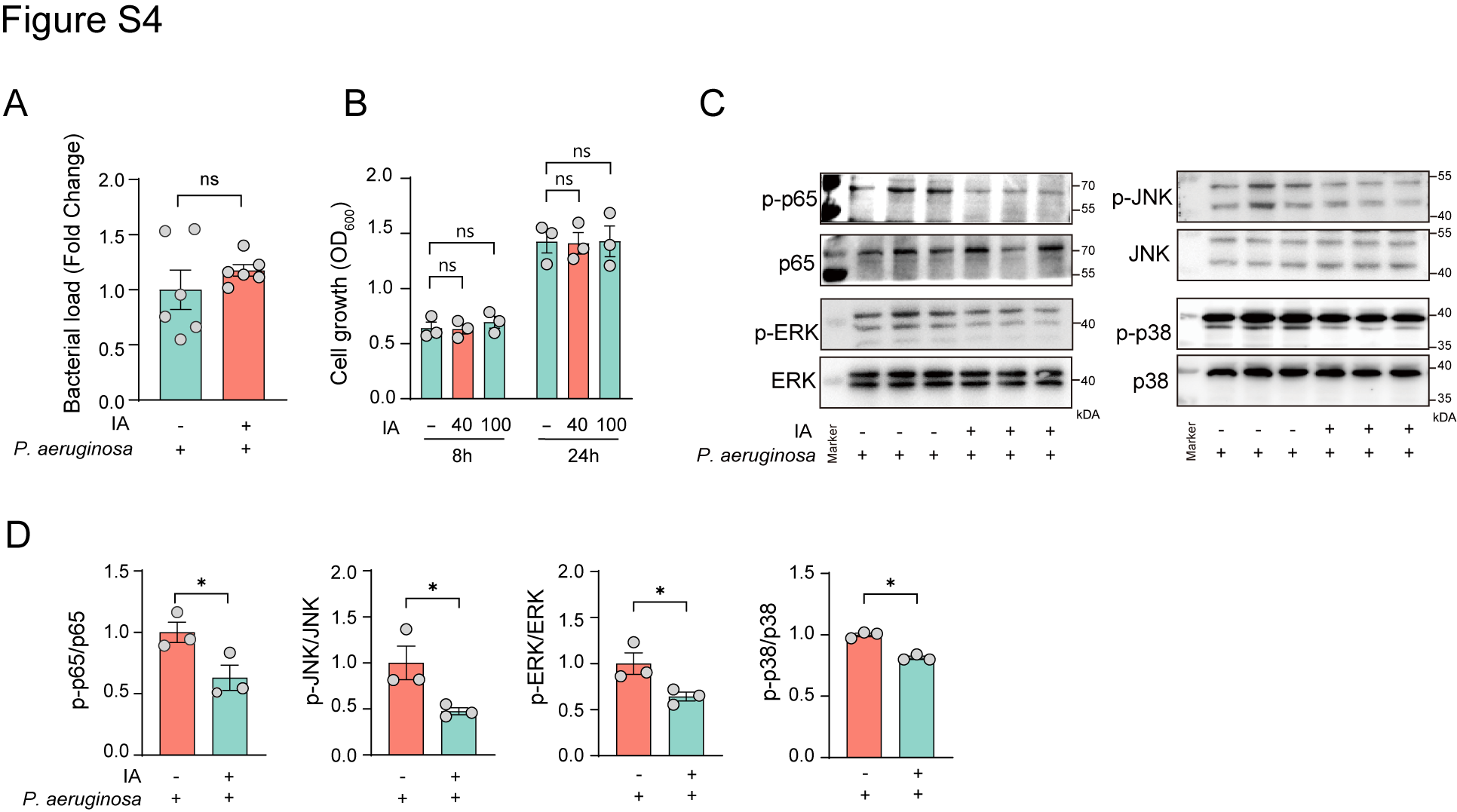


**Figure. S4. IA suppresses inflammatory pathways in alveolar macrophages without affecting bacterial growth. A** Bacterial load in the lungs following *P. aeruginosa* infection and IA (30mg/kg) treatment for 8 hours. n=6. **B** The OD_600_ values of *P. aeruginosa* co cultured with IA (40μM, 100uM) in vitro for 8 and 24 hours. n=3. **C, D** Protein expression levels in AMs of mice infected with *P. aeruginosa* for 6 hours under IA intervention. n=3. Data are shown as mean ± SEM. **p*<0.05 were assessed by a two-tailed unpaired Student's t-test.


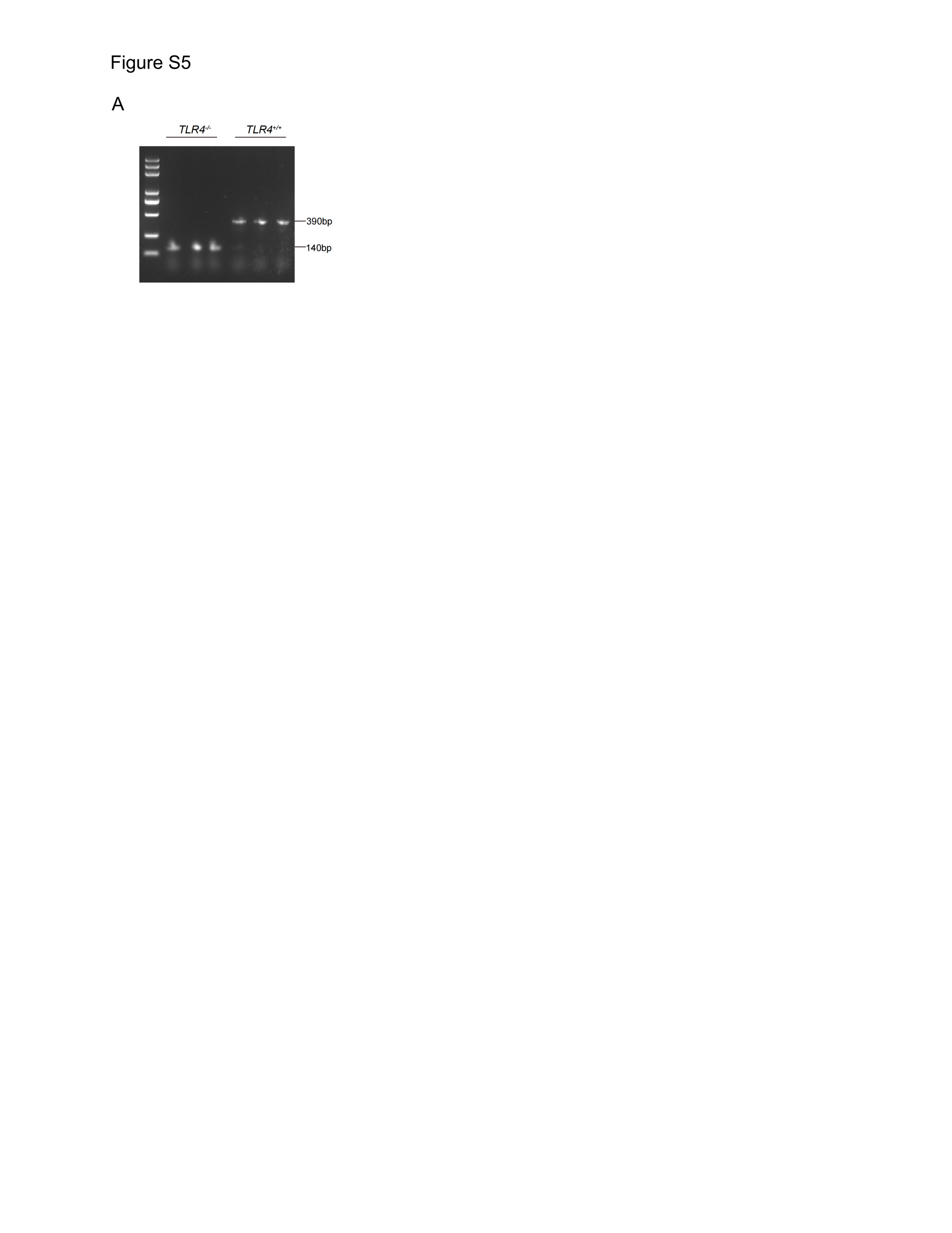


**Figure S5. Successful construction of *TLR4* knockout mice. A** Genotyping identification of *TLR4* knockout mice. *TLR4^-/-^* shows a 140 bp fragment, *TLR4^+/+^* shows a 390 bp fragment. n=3.

**Table S1. Clinical information of pneumonia patients**

| **Severe Pneumonia** | **Total (n=15)** |
| --- | --- |
| **Parameters** | No. of patients |
| **Gender** |  |
| Male (%) | 10 (66%) |
| Female (%) | 5 (33%) |
| **Age (years)** |  |
| Male (range) | 75.5 (50-85) |
| Female (range) | 69 (58-76) |
| **Co-morbidities** |  |
| Cerebral Infarction | 6 |
| Atrial Fibrillation | 4 |
| Chronic Obstructive Pulmonary Disease | 3 |
| Diabetes Mellitus | 4 |
| Hypertension | 4 |
| Sepsis | 3 |
| Hepatic Insufficiency | 2 |
| Renal Insufficiency | 2 |
| Multiple Organ Dysfunction Syndrome | 2 |
| Coronary Heart Disease | 1 |
| **Infection source** |  |
| *Pseudomonas aeruginosa* | 8 |
| *Klebsiella pneumoniae* | 5 |
| *Staphylococcus aureus* | 3 |
| *Acinetobacter baumannii* | 3 |
| *Enterococcus faecium* | 1 |
| *Stenotrophomonas maltophilia* | 1 |
| *Staphylococcus capitis* | 1 |
| *Haemophilus influenzae* | 1 |

**Table S2. Clinical information of non-pneumonia patients**

| **Non-Pneumonia** | **Total (n=15)** |
| --- | --- |
| **Parameters** | No. of patients |
| **Gender** |  |
| Male (%) | 10 (66%) |
| Female (%) | 5 (33%) |
| **Age (years)** |  |
| Male (range) | 63 (36-81) |
| Female (range) | 72 (59-76) |
| **Disease type** |  |
| Cerebral Hemorrhage | 6 |
| Fracture | 6 |
| Cerebral Infarction | 4 |
| Hypertension | 2 |
| Osteoporosis | 2 |
| Hyperuricemia | 2 |
| Diabetes Mellitus | 1 |

**Table S3. Primers for qPCR**

|  | **Forward primer (5**′**-3**′**)** | **Reverse primer (5**′**-3**′**)** |
| --- | --- | --- |
| *18S* | CGATCCGAGGGCCTCACTA | AGTCCCTGCCCTTTGTACACA |
| 16S | GTGSTGCAYGGYTGTCGTCA | ACGTCRTCCMCACCTTCCTC |
| *Tnf-α* | CAGGCGGTGCCTATGTCTC | CGATCACCCCGAAGTTCAGTAG |
| *Il-6* | CTGCAAGAGACTTCCATCCAG | AGTGGTATAGACAGGTCTGTTGG |
| *Il-1β* | GAAATGCCACCTTTTGACAGTG | TGGATGCTCTCATCAGGACAG |
| *S. salivarius* | GGCCGTCCTTCTTGTCCATA | AGAGGGCTGGAACAGTAGCA |
